# Liver-targeted *Angptl4* silencing by antisense oligonucleotide treatment attenuates hyperlipidaemia and atherosclerosis development in APOE*3-Leiden.CETP mice

**DOI:** 10.1093/cvr/cvae195

**Published:** 2024-09-11

**Authors:** Melanie Modder, Wietse In het Panhuis, Mohan Li, Salwa Afkir, Alexandra L Dorn, Amanda C M Pronk, Trea C M Streefland, Reshma A Lalai, Stefan Pierrou, Stefan K Nilsson, Gunilla Olivecrona, Sander Kooijman, Patrick C N Rensen, Milena Schönke

**Affiliations:** Division of Endocrinology, Department of Medicine, Leiden University Medical Center, Albinusdreef 2, 2333 ZA Leiden, The Netherlands; Einthoven Laboratory for Experimental Vascular Medicine, Leiden University Medical Center, Albinusdreef 2, 2333 ZA Leiden, The Netherlands; Division of Endocrinology, Department of Medicine, Leiden University Medical Center, Albinusdreef 2, 2333 ZA Leiden, The Netherlands; Einthoven Laboratory for Experimental Vascular Medicine, Leiden University Medical Center, Albinusdreef 2, 2333 ZA Leiden, The Netherlands; Division of Endocrinology, Department of Medicine, Leiden University Medical Center, Albinusdreef 2, 2333 ZA Leiden, The Netherlands; Einthoven Laboratory for Experimental Vascular Medicine, Leiden University Medical Center, Albinusdreef 2, 2333 ZA Leiden, The Netherlands; Division of Endocrinology, Department of Medicine, Leiden University Medical Center, Albinusdreef 2, 2333 ZA Leiden, The Netherlands; Einthoven Laboratory for Experimental Vascular Medicine, Leiden University Medical Center, Albinusdreef 2, 2333 ZA Leiden, The Netherlands; Division of Endocrinology, Department of Medicine, Leiden University Medical Center, Albinusdreef 2, 2333 ZA Leiden, The Netherlands; Einthoven Laboratory for Experimental Vascular Medicine, Leiden University Medical Center, Albinusdreef 2, 2333 ZA Leiden, The Netherlands; Division of Endocrinology, Department of Medicine, Leiden University Medical Center, Albinusdreef 2, 2333 ZA Leiden, The Netherlands; Einthoven Laboratory for Experimental Vascular Medicine, Leiden University Medical Center, Albinusdreef 2, 2333 ZA Leiden, The Netherlands; Division of Endocrinology, Department of Medicine, Leiden University Medical Center, Albinusdreef 2, 2333 ZA Leiden, The Netherlands; Einthoven Laboratory for Experimental Vascular Medicine, Leiden University Medical Center, Albinusdreef 2, 2333 ZA Leiden, The Netherlands; Division of Endocrinology, Department of Medicine, Leiden University Medical Center, Albinusdreef 2, 2333 ZA Leiden, The Netherlands; Einthoven Laboratory for Experimental Vascular Medicine, Leiden University Medical Center, Albinusdreef 2, 2333 ZA Leiden, The Netherlands; Lipigon Pharmaceuticals AB, Tvistevägen 48C, 907 36 Umeå, Sweden; Lipigon Pharmaceuticals AB, Tvistevägen 48C, 907 36 Umeå, Sweden; Lipigon Pharmaceuticals AB, Tvistevägen 48C, 907 36 Umeå, Sweden; Department of Medical Biosciences, Umeå University, Umeå, Sweden; Division of Endocrinology, Department of Medicine, Leiden University Medical Center, Albinusdreef 2, 2333 ZA Leiden, The Netherlands; Einthoven Laboratory for Experimental Vascular Medicine, Leiden University Medical Center, Albinusdreef 2, 2333 ZA Leiden, The Netherlands; Division of Endocrinology, Department of Medicine, Leiden University Medical Center, Albinusdreef 2, 2333 ZA Leiden, The Netherlands; Einthoven Laboratory for Experimental Vascular Medicine, Leiden University Medical Center, Albinusdreef 2, 2333 ZA Leiden, The Netherlands; Division of Endocrinology, Department of Medicine, Leiden University Medical Center, Albinusdreef 2, 2333 ZA Leiden, The Netherlands; Einthoven Laboratory for Experimental Vascular Medicine, Leiden University Medical Center, Albinusdreef 2, 2333 ZA Leiden, The Netherlands

**Keywords:** Hyperlipidaemia, Atherosclerosis, Angptl3, Angptl4, Antisense oligonucleotides

## Abstract

**Aims:**

Angiopoietin-like 3 (ANGPTL3) and 4 (ANGPTL4) inhibit lipoprotein lipase to regulate tissue fatty acid (FA) uptake from triglyceride (TG)-rich lipoproteins such as very low density lipoproteins (VLDL). While pharmacological inhibition of ANGPTL3 is being evaluated as a lipid-lowering strategy, systemic ANGPTL4 inhibition is not pursued due to adverse effects. This study aims to compare the therapeutic potential of liver-specific *Angptl3* and *Angptl4* silencing to attenuate hyperlipidemia and atherosclerosis development in APOE*3-Leiden.CETP mice, a well-established humanized model for lipoprotein metabolism.

**Methods and results:**

Mice were subcutaneously injected twice per week with saline or liver-targeted antisense oligonucleotides against *Angptl3*, *Angptl4*, both, or a scrambled oligonucleotide. Plasma lipid levels, VLDL clearance, and hepatic VLDL production were determined, and atherosclerosis development was assessed. For toxicological evaluation, cynomolgus monkeys were treated with three dosages of liver-targeted *ANGPTL4*-silencing oligonucleotides. Liver-targeted *Angptl4* silencing reduced plasma TGs (−48%) and total cholesterol (−56%), explained by higher VLDL-derived FA uptake by brown adipose tissue and lower VLDL production by the liver. Accordingly, *Angptl4* silencing reduced atherosclerotic lesion size (−86%) and improved lesion stability. Hepatic *Angptl3* silencing similarly attenuated hyperlipidemia and atherosclerosis development. While *Angptl3* and *Angptl4* silencing lowered plasma TGs in the refed and fasted state, respectively, combined *Angptl3/4* silencing lowered plasma TGs independent of the nutritional state. In cynomolgus monkeys, anti-*ANGPTL4* ASO treatment was well tolerated without adverse effects.

**Conclusion:**

Liver-targeted *Angptl4* silencing potently attenuates hyperlipidemia and atherosclerosis development in APOE*3-Leiden.CETP mice, and liver-targeted *ANGPTL4* silencing is well tolerated in non-human primates. These data warrant further clinical development of liver-targeted *ANGPTL4* silencing.

## Graphical Abstract

**Graphical Abstract:**
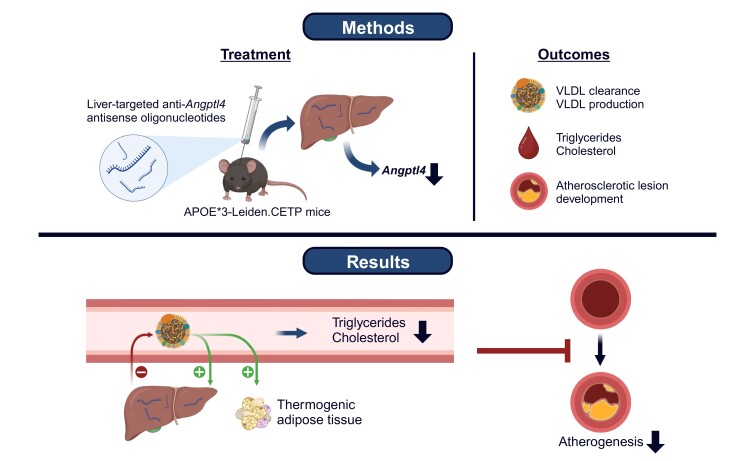

**Time for primary review: 12 days**

## 1. Introduction

Approximately one-third of all global deaths can be attributed to cardiovascular diseases (CVDs), with atherosclerosis as the main underlying pathology.^1^ Despite the current therapeutic strategies that effectively lower traditional risk factors including hypertension and LDL-cholesterol (LDL-C) levels, a considerable residual CVD event risk remains.^2^ A few years ago, circulating triglyceride (TG)-rich lipoproteins (TRLs) including chylomicrons and very low density lipoproteins (VLDL) were identified as a causal and independent risk factor for atherosclerotic (as)CVD.^3^ As more than 25% of the population has mild to moderately elevated plasma TG levels,^4^ there is a necessity for the development of pharmacological treatments that lower TG as well as LDL-C.

The predominant mechanism by which TRL-derived TGs are cleared from the circulation is extracellular lipoprotein lipase (LPL)-mediated hydrolysis, resulting in uptake of TG-derived fatty acids (FAs) by extrahepatic tissues and the generation of cholesterol-enriched TRL remnants that are cleared by the liver.^5^ The significance of LPL activity for the regulation of plasma TG levels is demonstrated by observations that human carriers of loss-of-function mutations in the *LPL* gene have elevated plasma TG levels, with a corresponding increased CVD risk.^6,7^*LPL* is expressed by many types of cells, but predominantly by adipocytes, myocytes, and macrophages, and the activity of the encoded protein is regulated by numerous factors that respond to different nutritional states. Among others, angiopoietin-like 3 (ANGPTL3) and angiopoietin-like 4 (ANGPTL4) are important inhibitors of LPL that antagonize TRL delipidation.^8,9^ Although both ANGPTL3 and ANGPTL4 suppress LPL activity, their expression patterns and specific functions in TG metabolism differ. *ANGPTL3* is almost exclusively expressed by hepatocytes in a constant manner and the protein is secreted into the circulation.^10^ Nonetheless, ANGPTL3 mainly inhibits LPL in skeletal muscle and cardiac tissue in the postprandial state through interaction with ANGPTL8 that is produced postprandially by the liver and other organs, and strongly amplifies the LPL-inhibitory capacity of ANGPTL3.^11^ In contrast, ANGPTL4 is expressed in a variety of tissues and inhibits LPL activity predominantly during fasting when its gene expression is elevated. Therefore, LPL activity in white adipose tissue (WAT) is low during fasting, preventing TRL-derived TG storage.^9,12^ In thermogenic brown adipose tissue (BAT), ANGPTL4 dictates diurnal and cold-induced LPL activity to replenish intracellular lipid stores following sustained thermogenesis.^13,14^

Since the discovery that human loss-of-function gene variants of *ANGPTL3* and *ANGPTL4* associate with lowered plasma TG levels and reduced CVD risk,^15,16^ therapeutic silencing of these LPL inhibitors has received much attention.^17^ Monoclonal antibodies (mAbs) and antisense oligonucleotides (ASOs) targeting ANGPTL3 have been successfully shown to reduce plasma TG and LDL-C in mice and humans.^18–22^ In contrast, global inhibition of ANGPTL4 is not considered a viable therapeutic strategy due to adverse effects such as the formation of lipogranulomatous lesions in the intestines and chylous ascites, likely as a result of the heterogeneous expression and function of ANGPTL4 across tissues.^23–25^ Tissue-specific ANGPTL4 targeting, however, is a promising approach that circumvents these adverse effects. For example, genetic deletion of *Angptl4* in adipose tissue safely attenuates hyperlipidemia and atherosclerosis development,^26^ but adipose-targeted drug delivery is currently not possible in humans. In contrast, hepatocyte-targeted drug delivery is possible using ASOs with a linked triantennary *N*-acetyl galactosamine (GalNAc) cluster, which is recognized with high affinity by the hepatocyte-specific asialoglycoprotein receptor.^27^ In line with high expression and secretion of ANGPTL4 by the liver, hepatic silencing of ANGPTL4 has been shown to effectively and safely attenuate hyperlipidemia.^28,29^

To investigate whether liver-targeted *Angptl4* suppression holds similar therapeutic potential as *Angptl3* inhibition in lowering hyperlipidemia and atherosclerosis development, we compare ASO-mediated liver-targeted silencing of *Angptl3* and *Angptl4* alone and combined in APOE*3-Leiden.CETP mice, a well-established humanized mouse model for lipoprotein metabolism and cardiometabolic diseases. We provide the proof of concept that liver-targeted anti-*Angptl4* ASO treatment strongly attenuates hyperlipidemia and atherosclerosis development, and combined *Angptl3/4* silencing has added lipid-lowering benefit. We furthermore show that the administration of anti-*ANGPTL4* ASOs in non-human primates is safe without any adverse events.

## 2. Methods

### 2.1 Mouse experiments

APOE*3-Leiden.CETP mice were generated as previously described.^30–33^ Female APOE*3-Leiden.CETP mice of 8–14 weeks of age were housed under standard conditions in groups of 3–4 mice per cage in a 12:12 h light/dark cycle with *ad libitum* access to a Western-type diet (16% fat, 0.15% cholesterol; Diet T; ssniff-Spezialdiäten GmbH, Soest, Germany, see supplement for detailed composition) and water unless stated otherwise. After a dietary run-in period of 3 weeks, baseline measurements were taken and the mice were block-randomized into five intervention groups that were balanced for 4 h fasted plasma TG and total plasma cholesterol (TC), age, body weight, and lean and fat body mass (EchoMRI 100-Analyzer; EchoMRI, Houston, TX, USA).

In Experiment 1, mice (*n* = 16 per group) were subcutaneously injected twice per week with saline solution, a liver-targeted negative (scrambled) ASO (1.25 mg/kg), anti-*Angptl3* ASO (1.25 mg/kg), anti-*Angptl4* ASO (1.25 mg/kg), or anti-*Angptl3* ASO + anti-*Angptl4* ASO (1.25 mg/kg per ASO) that were provided by Lipigon Pharmaceuticals AB (Umeå, Sweden). During the last week of the study, the mice were individually housed in calorimetric home cages. After 2 weeks, body weight, body composition, and food intake were measured. Then, the mice were fasted for 4 h before assessing plasma lipid levels as well as organ uptake of TRL-mimicking particles (*n* = 8 per group) or hepatic VLDL production (*n* = 8 per group) around *Zeitgeber* time (ZT)6. Livers from the mice in which organ uptake of lipids was assessed were used for gene expression analyses, histology, and lipid and water content measurements (*n* = 8 per group). WAT from these mice was used for LPL protein abundance and activity measurements.

In Experiment 2, mice (*n* = 16 per group) were subcutaneously injected with saline, a liver-targeted negative (scrambled) ASO (0.60 mg/kg), anti-*Angptl3* ASO (0.30 mg/kg), anti-*Angptl4* ASO (0.30 mg/kg), or anti-*Angptl3* ASO + anti- *Angptl4* ASO (0.30 mg/kg per ASO) twice per week for a total of 12 weeks. Body weight, body composition, food intake, and 4 h fasted plasma lipid levels were monitored throughout the intervention. At the end of the study, eight mice per group were killed after 16 h of fasting and the other eight mice per group after 12 h of fasting followed by 4 h of refeeding to measure plasma lipid levels in different nutritional states. All animal experiments were conducted in accordance with the Institute for Laboratory Animal Research Guide for the Care and Use of Laboratory Animals and were approved by the National Committee for Animal Experiments and by the Ethics Committee on Animal Care and Experimentation of the Leiden University Medical Center, The Netherlands.

### 2.2 Gene expression analysis

RNA was isolated from frozen liver (∼30 mg) using TriPure RNA Isolation Reagent (11667165001, Sigma-Aldrich, St. Louis, USA) and a FastPrep-24™ 5G bead beating grinder and lysis system (4.0 m/s for 10 s; MP Biomedicals™, Santa Ana, CA, USA). cDNA was synthesized from 1 µg RNA using M-MLV Reverse Transcriptase (M1705, Promega, Madison, WI, USA) and qPCR was conducted utilizing SYBR green kit (Promega) and a CFX96 PCR machine (Bio-Rad, Hercules, USA). Gene expression was normalized to *β2-microglobulin* and expressed relative to the saline group. Primer sequences are displayed in Supplementary material online, *Table S1*.

### 2.3 Energy expenditure calculation

Cumulative energy expenditure over the duration of Experiment 1 was calculated using the energy balance technique that takes the cumulative metabolizable energy intake and the change in body composition into account as described by Ravussin *et al*.^34^

### 2.4 Indirect calorimetry

Indirect calorimetry was carried out in metabolic home cages (Sable Systems International, Las Vegas, NV, USA). The mice were single-housed and data collection started after 2 days of acclimatization. Specifically, O_2_ consumption (mL/h) and CO_2_ production (mL/h) were recorded, from which energy expenditure, fat oxidation rate, and carbohydrate oxidation rate were calculated as described previously.^35^ Data are shown as whole-body oxidation rates.

### 2.5 Plasma measurements

Plasma TG and TC levels were measured using Cobas Triglycerides (106571) or Cobas Total Cholesterol (106570) enzymatic reagents (Roche Diagnostics, Mannheim, Germany). Precisely, 200 μL reagent (the reagent was undiluted for TG and 3× diluted for TC) was added to 7.5 μL plasma sample (5× diluted) and incubated at room temperature for 30 min prior to measuring transmittance at 492 vs. 650 nm (for TG) or at 505 vs. 650 nm (for TC). To calculate total TC exposure, the area under the curve was determined from regularly monitored plasma TC levels over time. For quantification of HDL cholesterol (HDL-C), apolipoprotein B (ApoB) containing lipoproteins were precipitated from plasma by adding 20% polyethylene glycol in 200 mM glycine-buffered saline (pH 10), and TC was measured in the supernatant as described previously. Non-HDL-C plasma levels were calculated by subtracting plasma HDL-C from plasma TC. Plasma alanine aminotransferase (ALT) activity was determined by the Alanine Aminotransferase Activity Assay Kit (Sigma-Aldrich) according to the manufacturer’s protocol. Circulating levels of IFN-γ, IL-1β, and TNF-α were measured in plasma samples collected at the end of Experiment 1 using a customized multiplex U-Plex Biomarker Group 1 (mouse) Kit (Meso Scale Discovery; Rockville, MD, USA) according to the manufacturer’s protocol.

### 2.6 *In vivo* organ uptake of TRL-mimicking particles

TRL-mimicking particles labelled with glycerol tri[^3^H]oleate and [^14^C]cholesteryl oleate were prepared as described previously.^36^ The particles were injected into the tail vein (1 mg TG per mouse in 200 μL saline) 15 min before the mice were killed via CO_2_ asphyxiation. Subsequently, the mice were perfused transcardially with ice-cold PBS for 5 min before various (parts of) organs (∼50–150 mg) were collected and dissolved in 0.5 mL Solvable (6NE9100, PerkinElmer, Waltham, USA) at 56°C overnight, after which 5.0 mL Ultima Gold (6013329, PerkinElmer) was added. ^3^H-activity and ^14^C-activity were measured simultaneously with a scintillation counter (Tri-Carb 2910 TR, PerkinElmer) and expressed as a percentage of the injected dose per whole tissue. Lipolysis index was calculated as the ratio between ^3^H-activity and ^14^C-activity per tissue.

### 2.7 Hepatic VLDL-TG and VLDL-ApoB production assay

The mice were anaesthetized by intraperitoneal injection with a combination of ventranquil (6.25 mg/kg), midazolam (6.25 mg/kg), and fentanyl (0.31 mg/kg) in a total volume of 8 μL/g body weight, followed by subcutaneous injections of 50 μL every 45 min to sustain anaesthesia. To label newly synthesized ApoB, the mice were then injected intravenously with 10 μCi Tran[^35^S] label (IS-103; Hartmann Analytic, Braunschweig, Germany). After 30 min, the mice were injected intravenously with 10% Triton WR-1339 (5 μL/g body weight, Sigma-Aldrich) to block LPL activity and thus VLDL-TG clearance. Blood (∼20 µL per time point) was collected via the tail vein prior to and 15, 30, 60, and 90 min after the Triton WR-1339 injection for quantification of plasma TG levels as described previously. At 120 min, the mice were exsanguinated via the eye. Plasma was ultracentrifuged in a density gradient, after which VLDL was isolated by aspiration.^37^^35^S-activity was measured in the acquired VLDL with and without the addition of isopropanol in a 1:1 ratio to precipitate ApoB, and ^35^S-activity was expressed as disintegrations per minute per mL, and VLDL-TG and VLDL-TC were measured as described previously. A colorimetric assay was used to determine phospholipids (PL) (INstruchemie, Delfzijl, The Netherlands) and protein content (Thermo Fisher Scientific, Waltham, USA) of the particles.

### 2.8 Liver histology, lipid, and water content

Formaldehyde-fixated, paraffin-embedded liver was cross-sectioned into 5 μm thick sections. One section was stained with haematoxylin and eosin, one section was stained for the macrophage marker F4/80 as described previously,^38^ and in another section collagen was stained using Direct Red and Fast Green (both 1:1000; 365548-5G and F7258S, respectively; Sigma-Aldrich). F4/80 and collagen content were quantified using ImageJ software (version 1.52a, National Institutes of Health, Bethesda, MD, USA). Lipids were extracted from frozen liver tissue using a modified version of the Bligh and Dyer protocol,^39^ and TG and TC levels were measured as described previously. To determine water content of the liver, 50–150 mg tissue was weighed prior to and after freeze-drying for 5 days (Alpha 1-4 LSCbasic, Martin Christ, Osterode am Harz, Germany).

### 2.9 LPL protein abundance and activity

LPL protein abundance was measured in protein lysates from gWAT. For this, proteins were separated by SDS-PAGE using Criterion XT Precast gels (Bio-Rad, Hercules, CA, USA) and transferred to the polyvinylidene difluoride membrane (Immobilon-P; Millipore, Billerica, MA, USA). Total protein on the membrane was stained with Ponceau S and the membrane was washed, blocked, and incubated overnight with the primary goat anti-mouse LPL antibody (kindly provided by Sander Kersten^29^). Following the incubation with a horseradish peroxidase-conjugated secondary antibody (rabbit anti-goat; Bio-Rad), bands were visualized using enhanced chemiluminescence. The bands were quantified and normalized to the Ponceau S staining. Representative blots are shown in the supplement (see Supplementary material online, *Figure S2*). LPL activity was measured in gWAT as described previously.^14^

### 2.10 Atherosclerosis quantification

Formaldehyde-fixated, paraffin-embedded hearts were cross-sectioned (5 μm) perpendicular to the axis of the aorta throughout the aortic root area. The sections were stained with haematoxylin–phloxine–saffron for histological analysis of the lesion area as described previously.^31^ Four subsequent sections (50 μm apart) were analysed per valve starting from the first appearance of the open aortic valve leaflets. The lesions were categorized by severity (mild: Types I–III or severe: Types IV and V) according to the guidelines of the American Heart Association adapted for mice.^40^ Quantification of macrophage content was performed using rat monoclonal anti-mouse MAC-3 antibody (1:1000; 550292; BD Pharmingen, San Diego, USA) and secondary goat anti-rat IgG (MP7444; Vector Laboratories Inc., Burlingame, USA). Monoclonal mouse antibody (1:1000; M0851; Dako, Heverlee, The Netherlands) against smooth muscle cell actin and secondary goat anti-mouse IgG (1:400; K4003; Dako) were used to quantify smooth muscle cells. The immunoperoxidase complexes on the secondary antibodies were visualized with Nova Red (SK-4800, Vector Laboratories Inc.) and Liquid Dab + Substrate Chromogen System (K3468, Dako) for the macrophage and smooth muscle cell quantification, respectively. A solution of direct red and fast green (both 1:1000; 365548-5G and F7258S, respectively; Sigma-Aldrich) was used to stain collagen. The total lesion area and composition were determined using Image J software (version 1.52a, National Institutes of Health). Lesion stability index was calculated per mouse by dividing the sum of relative collagen- and smooth muscle cell-positive lesion area by relative macrophage-positive area.

### 2.11 Toxicology study in cynomolgus monkeys

The safety of a liver-targeted ASO designed to inhibit human *ANGPTL4* mRNA was assessed in 1–2 year old cynomolgus monkeys (*Macaca fascicularis*) at Charles River Laboratories (Tranent, UK). The monkeys were group-housed, had *ad libitum* access to water, and were fed with a balanced diet (Special Diet Services Mazuri Primate Diet) enriched with fruits, vegetables, nuts, and seeds. For a period of 4 weeks, the animals received weekly subcutaneously injections with saline (*n* = 5 males and *n* = 5 females), 3 mg/kg anti-*ANGPTL4* ASO (*n* = 3 males and *n* = 3 females), 10 mg/kg anti-*ANGPTL4* ASO (*n* = 3 males and *n* = 3 females), or 30 mg/kg anti-*ANGPTL4* ASO (*n* = 5 males and *n* = 5 females). The monkeys weighed 2.1–3.4 kg. After the treatment period, three males and three females per group were euthanized for toxicological evaluation. In addition, two males and two females from the group of monkeys treated with saline or 30 mg/kg anti-*ANGPTL4* ASO were euthanized after a 4-week washout period. The animal procedures were performed in accordance with the Organization for Economic Co-operation and Development Principles of Good Laboratory Practice as incorporated into the United Kingdom Statutory Instrument for GLP. The full toxicology report is available upon request.

### 2.12 Statistical analysis

Data were tested for normality, and in case of normal distributions, statistical analyses between groups were performed by one-way ANOVA with *post hoc* Šídák’s test. In the VLDL production experiment, one-way ANOVA with *post hoc* Šídák’s test was performed on calculated slopes of TG. In case of non-normal distributions, Kruskal–Wallis tests with a *post hoc* Dunn’s test were used instead of one-way ANOVA. Two-way ANOVA or mixed-effects models with *post hoc* Tukey’s test were used for repeated measurement in case of sequential sampling and for the fasting-feeding measurements. When data of repeated measurements were not normally distributed, two-way ANOVA tests were performed on ranked values. Additional two-way ANOVA was used in which the saline group was excluded to test for the main and interaction effects of anti-*Angptl3* and anti-*Angptl4* ASO treatments. Pearson correlation was used to calculate correlation coefficients. Statistical outliers were removed using Grubb’s test. Statistical analyses were performed with GraphPad Prism software, version 9.3.1 (GraphPad, La Jolla, CA, USA). The value of *P* < 0.05 was considered statistically significant. Data are presented as means ± SEM.

## 3. Results

### 3.1 Liver-targeted *Angptl3* and *Angptl4* silencing strongly lowers plasma lipid levels

**Figure 1:**
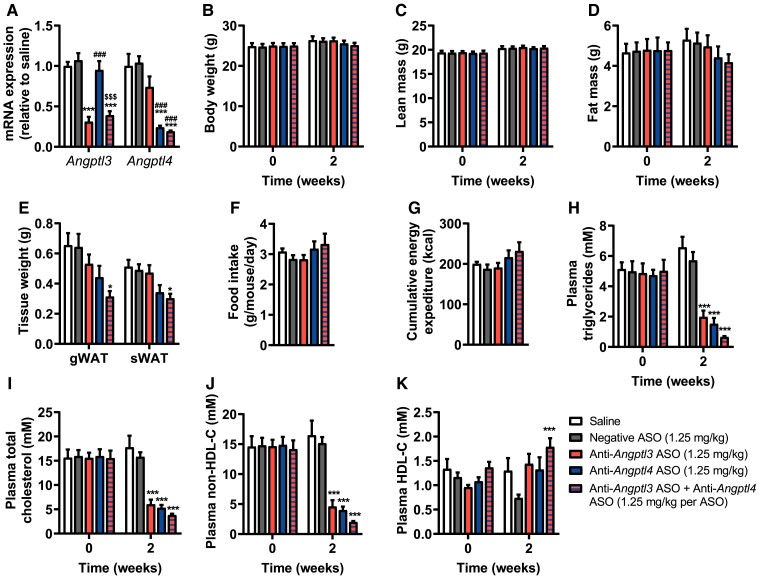
Short-term liver-targeted *Angptl3* and *Angptl4* silencing alleviates hyperlipidaemia in APOE*3-Leiden.CETP mice. Over a period of 2 weeks, female APOE*3-Leiden.CETP mice (8–14 weeks of age at the start of the study) were injected five times with saline (white) or the following liver-targeted ASOs: negative ASO (scrambled; 1.25 mg/kg, grey), anti-*Angptl3* ASO (1.25 mg/kg, red), anti-*Angptl4* ASO (1.25 mg/kg, blue), or anti-*Angptl3* ASO + anti-*Angptl4* ASO (1.25 mg/kg per ASO, red–blue striped). At the endpoint, hepatic (*A*) expression of *Angptl3* and *Angptl4* was measured (*n* = 6–8 per group). At weeks 0 and 2, (*B*) body weight, (*C*) lean mass, and (*D*) fat mass were determined (*n* = 8 per group). (*E*) Gonadal white adipose tissue (gWAT) and subcutaneous (s)WAT were weighed (*n* = 8 per group). During the last week of the experiment, (*F*) food intake was monitored while mice were single-housed (*n* = 6 per group). (*G*) Cumulative energy expenditure was calculated based on the metabolizable energy intake and changes in fat mass and lean mass. At weeks 0 and 2, levels of plasma (*H*) triglycerides, (*I*) total cholesterol, (*J*) non-HDL-C, and (*K*) HDL-C were measured after 4 h of fasting (*n* = 7–8 per group). Values are presented as means ± SEM. All groups were compared with each other, but the comparison with the saline group is not shown. ^*^ASO group vs. negative ASO; ^#^ASO group vs. anti-*Angptl3* ASO; ^$^anti-*Angptl4* ASO vs. anti-*Angptl3* ASO + anti-*Angptl4* ASO. ^*^P ≤ 0.05, ^**^P ≤ 0.01, ^***,###,$$$^*P* ≤ 0.001 according to one-way ANOVA (*A*, *E*, *F*, *G*), two-way ANOVA (*B–D*, *H*, *I*), or mixed-effects analysis (*J*, *K*) and following Tukey’s (*A–I*) or Šídák’s (*J*, *K*) multiple comparison test, respectively.

To compare the effects of liver-targeted *Angptl3* and *Angptl4* silencing alone and in combination on hyperlipidaemia, female APOE*3-Leiden.CETP mice were treated with saline, negative (scrambled) ASO, anti-*Angptl3* ASO, anti-*Angptl4* ASO, or anti-*Angptl3* ASO + anti-*Angptl4* ASO for a total duration of 2 weeks. Suppression of hepatic *Angptl3* and *Angptl4* expression was confirmed (*Angptl3* down-regulation of 71 and 64% by *Angptl3* and combined *Angptl3/4* silencing, respectively, and *Angptl4* down-regulation of 73 and 82% by *Angptl4* and combined *Angptl3/4* silencing, respectively) (*Figure 1A*). In non-hepatic tissues that express *Angptl4*, such as adipose tissue, the expression was not changed by the ASO treatment (see Supplementary material online, *Figure S1A*). While body weight (*Figure 1B*), lean mass (*Figure 1C*), and fat mass (*Figure 1D*) were unchanged, combined *Angptl3/4* silencing reduced gonadal WAT (gWAT) and subcutaneous WAT (sWAT) weights (*Figure 1E*) without affecting food intake (*Figure 1F*). This resulted in a slightly increased cumulative energy expenditure with the silencing of *Angptl4* and *Angptl3/4* as calculated from the calorie intake and body composition change throughout the study, which was, however, not statistically significant and not detectable with indirect calorimetry (*Figure 1G*, Supplementary material online, *Figure S1B*–*D*). Nonetheless, *Angptl4* and *Angptl3/4* silencing significantly increased the fat oxidation during the dark period (see Supplementary material online, *Figure S1C*). Both *Angptl3* and *Angptl4* silencing strongly lowered plasma TG (*Figure 1H*), TC (*Figure 1I*), and non-HDL-C levels (*Figure 1J*) compared with negative ASO treatment. The reductions in plasma TG, TC, and non-HDL-C levels by combined *Angptl3/4* silencing were comparable to those induced by the single ASO treatments. In contrast, only combined *Angptl3/4* silencing increased plasma HDL-C levels compared with negative ASO treatment (*Figure 1K*).

### 3.2 Liver-targeted *Angptl4* silencing increases lipid uptake by BAT and reduces hepatic VLDL production

**Figure 2:**
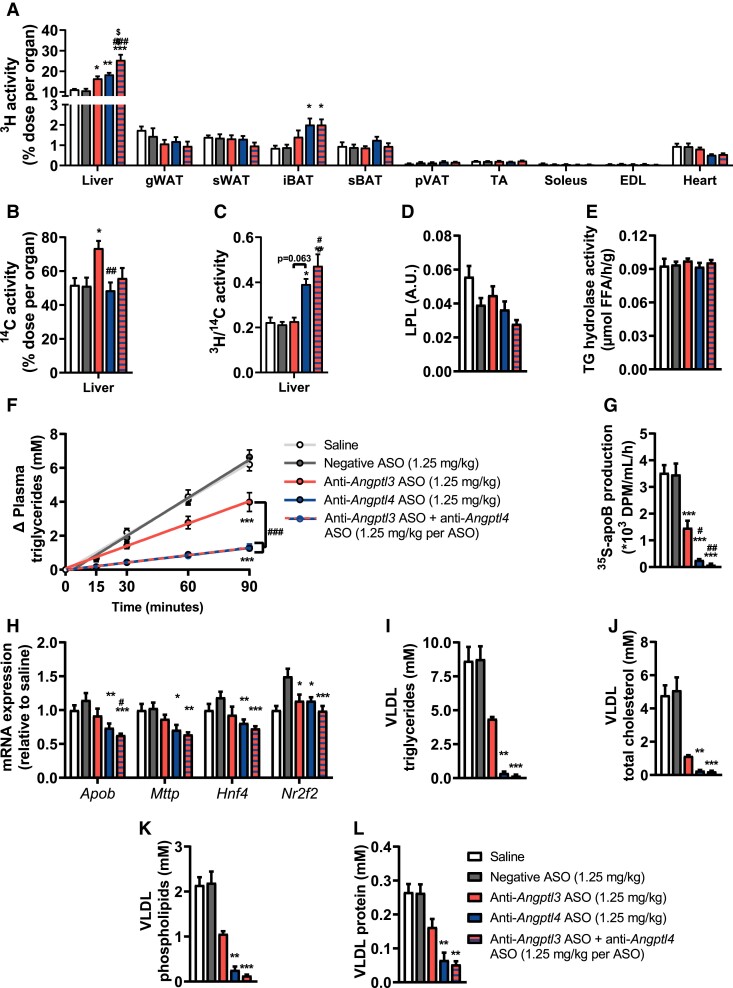
Liver-targeted Angptl4 silencing elevates lipid uptake by brown adipose tissue and liver while reducing hepatic VLDL production in APOE*3-Leiden.CETP mice. Over a period of 2 weeks, female APOE*3-Leiden.CETP mice (8–14 weeks of age at the start of the study) were injected five times with saline (white) or the following liver-targeted antisense oligonucleotides (ASO): negative ASO (scrambled; 1.25 mg/kg, grey), anti-*Angptl3* ASO (1.25 mg/kg, red), anti-*Angptl4* ASO (1.25 mg/kg, blue), or anti-*Angptl3* ASO + anti-*Angptl4* ASO (1.25 mg/kg per ASO, red–blue striped). At the end of the treatment period, half of the mice were injected with triglyceride (TG)-rich lipoprotein-like particles double-labelled with glycerol tri[^3^H]oleate and [^14^C]cholesteryl oleate to assess uptake of (*A*) [^3^H]oleate and (*B*) [^14^C]cholesteryl oleate by the liver, gonadal white adipose tissue (gWAT), subcutaneous (s)WAT, interscapular brown adipose tissue (iBAT), subscapular (s)BAT, perivascular (pV)AT, tibialis anterior (TA) muscle, soleus, extensor digitorum longus (EDL), and heart (*n* = 7–8 per group). (*C*) The ratio of ^3^H-activity over ^14^C-activity is shown for the liver (*n* = 7 per group). In gWAT, (*D*) lipoprotein lipase protein abundance and (*E*) lipolytic activity were determined (*n* = 4–8 per group). At the endpoint, the other half of the mice were injected with Tran[^35^S] label followed by Triton WR1339, and the TG levels were determined in plasma samples drawn at the indicated time points, which were (*F*) plotted as the increase in TG to baseline, from which VLDL-TG production rate was determined by linear regression. (*G*) ApoB production was determined by measuring ^35^S in isolated VLDL (*n* = 6–8 per group). Hepatic (*H*) gene expression of *ApoB*, *Mttp*, *Hnf4*, and *Nr2f2* was measured (*n* = 6–8 per group). Concentrations of (*I*) TG, (*J*) total cholesterol, (*K*) phospholipids, and (*L*) protein within VLDL particles were measured and corrected for the total volume of isolated VLDL (*n* = 6–8 per group). Values are presented as means ± SEM. All groups were compared with each other, but the comparison with the saline group is not shown. ^*^any ASO group vs. negative ASO; ^#^any ASO group vs. anti-*Angptl3* ASO; ^$^anti-*Angptl4* ASO vs. anti-*Angptl3* ASO + anti-*Angptl4* ASO ^*,#^*P* < 0.05; ^**,##,$$^*P* < 0.01; ^***,###^*P* < 0.001, according to one-way ANOVA (*A–H*) or (non-parametric) Kruskal–Wallis test (*I–L*) and following Tukey’s (*A–H*) or (non-parametric) Dunn’s (*I–L*) multiple comparison test.

Next, we investigated if liver-targeted silencing of *Angptl3* and *Angptl4* reduced plasma lipid levels by similar or different mechanisms. Firstly, 4 h-fasted mice were injected with TRL-mimicking particles that were double-labelled with glycerol tri[^3^H]oleate and [^14^C]cholesteryl oleate, reflecting TG-derived FA and TRL-remnant uptake, respectively. *Angptl4* silencing and combined *Angptl3/4* silencing elevated [^3^H]oleate uptake by interscapular brown adipose tissue (iBAT) and liver, while hepatic [^14^C]cholesteryl oleate uptake was unaffected, thereby increasing the ^3^H/^14^C uptake ratio in the liver (*Figure 2A–C*). To investigate whether this was explained by an increased hepatic influx of [^3^H]oleate derived from peripheral lipolysis, we quantified the abundance of LPL protein in gWAT as well as its lipolytic activity *in vitro* but did not detect differences between the groups (*Figure 2D* and *E*, Supplementary material online, *Figure S2*). *Angptl3* silencing elevated hepatic [^3^H]oleate and [^14^C]cholesteryl oleate uptake alike (*Figure 2A* and *B*).

Secondly, we evaluated the VLDL-TG production rate as well as VLDL-ApoB production *in vivo* after 2 weeks of ASO treatment (1.25 mg/kg). Interestingly, both *Angptl3* and *Angptl4* silencing reduced hepatic TG production with a corresponding reduction in VLDL-[^35^S]ApoB production (*Figure 2F* and *G*). These reductions were stronger with *Angptl4* and combined *Angptl3/4* silencing compared with *Angptl3* silencing, and accompanied by significant reductions in hepatic expression of *Apob* and microsomal TG transfer protein (*Mttp*), genes involved in VLDL assembly, and their transcription factors *Hnf4a* and *Nr2f2* (*Figure 2H*). Likewise, all treatments decreased TG, TC, PL, and protein content within the VLDL fraction, while these reductions were the largest and only significant with *Angptl4* and combined *Angptl3/4* silencing (*Figure 2I–L*).

### 3.3 Short-term liver-targeted *Angptl3* and *Angptl4* silencing does not induce hepatic inflammation

Unexpectedly, *Angptl3*, *Angptl4*, and combined *Angptl3/4* silencing over 2 weeks of ASO treatment (1.25 mg/kg) increased liver weight (see Supplementary material online, *Figure S3A*) compared with negative ASO treatment. Although ASO-related toxicity is common in mice and often manifests in liver injury and inflammation,^41^ hallmarks of ASO toxicity,^41,42^ including the presence of inflammatory infiltrates, formation of basophilic granules upon ASO accumulation, signs of apoptosis, necrosis, or fibrosis, and an increased expression of genes encoding pro-inflammatory factors were not observed (see Supplementary material online, *Figure S3B*–*E*). Furthermore, whereas ASO-related hepatotoxicity can elevate plasma ALT activity 50- to 200-fold to up to 1000 U/L in mice,^42^ anti-*Angptl4* and combined ASO treatment only non-significantly induced a three-fold elevation in plasma ALT activity up to 60 U/L (see Supplementary material online, *Figure S3F*), indicating no severe liver damage. In line, the circulating levels of the pro-inflammatory cytokines IFN-γ, IL-1β, and TNF-α were not significantly elevated with the ASO treatment (see Supplementary material online, *Figure S3G*–*I*). Importantly, a 4-week toxicology study in cynomolgus monkeys showed that anti-*ANGPTL4* ASO treatment (weekly subcutaneous injections of up to 30 mg/kg) was well tolerated and did not induce any adverse effects, such as increased liver weight, abnormal hepatic microscopic findings, or elevated plasma levels of ALT and aspartate transaminase (AST) (see Supplementary material online, *Tables S2–S4*). The lowest ASO dose in this toxicology study exceeded the highest dosages used in the present mouse study (0.3–1.25 mg/kg) and in the successfully completed Phase I clinical trial (36 mg) 8- to 20-fold, respectively.

### 3.4 Liver-targeted *Angptl3* and *Angptl4* silencing lowers plasma TG in refed and fasted state, respectively

**Figure 3:**
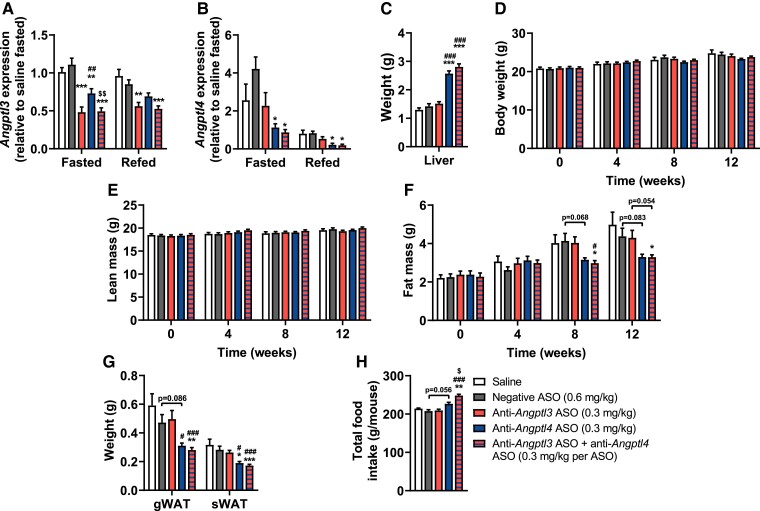
Long-term liver-targeted *Angptl4* silencing lowers adiposity in APOE*3-Leiden.CETP mice. Over a period of 12 weeks, female APOE*3-Leiden.CETP mice (8–14 weeks of age at the start of the study) were injected with saline (white) or the following liver-targeted antisense oligonucleotides (ASO): negative ASO (scrambled; 1.25 mg/kg, grey), anti-*Angptl3* ASO (0.3 mg/kg, red), anti-*Angptl4* ASO (0.3 mg/kg, blue), or anti-*Angptl3* ASO + anti-*Angptl4* ASO (0.3 mg/kg per ASO, red–blue striped). At the endpoint, hepatic expression of (*A*) *Angptl3* and (*B*) *Angptl4* were measured (*n* = 7–8 per group) and (*C*) livers were weighed (*n* = 16 per group). Every 4 weeks, (*D*) body weight, (*E*) lean mass, and (*F*) fat mass were measured (*n* = 15–16 per group). At the endpoint, (*G*) gonadal white adipose tissue (gWAT) and subcutaneous (s)WAT were weighed (*n* = 15–16 per group). (*H*) Food intake was monitored and expressed as total per mouse (*n* = 4 cages of three to four mice per cage). Values are presented as means ± SEM. All groups were compared with each other, but the comparison with the saline group is not shown. ^*^any ASO group vs. negative ASO; ^#^any ASO group vs. anti-*Angptl3* ASO; ^$^any group vs. anti-*Angptl4*. ^*,#,$^*P* < 0.05; ^**^P < 0.01; ^***,###^*P* < 0.001, according to two-way ANOVA (*A, B, D, E, H*), one-way ANOVA (*C*), two-way ANOVA on ranked values (*F*), or (non-parametric) Kruskal–Wallis test (*G*), and following, Šídák’s (*C*), Tukey’s (*A, B, D, E, F, H*), or (non-parametric) Dunn’s (*G*) multiple comparison test, respectively.

Since short-term liver-targeted *Angptl3* and *Angptl4* silencing potently attenuated hyperlipidemia, we next assessed to what extent these treatments would also reduce atherosclerosis development over a treatment period of 12 weeks. To minimize hepatic enlargement, we first tested various lower ASO concentrations for their potency to down-regulate hepatic *Angptl3* and *Angptl4* expression (data not shown). We accordingly reduced the dosage from 1.25 to 0.30 mg/kg, with the exception of the negative ASO (0.60 mg/kg) to match the total amount of ASO administered to the combined group (0.30 mg/kg of both anti-*Angptl3* and anti-*Angptl4* ASO). Suppression of hepatic *Angptl3* and *Angptl4* expression by the ASO treatments at this dose was confirmed (*Figure 3A* and *B*). *Angptl3* silencing and combined *Angptl3/4* silencing down-regulated *Angptl3* expression by 58 and 56% in the fasted state, and by 35 and 38% in the refed state, respectively (*Figure 3A*). *Angptl4* silencing and combined *Angptl3/4* silencing down-regulated *Angptl4* expression by 75 and 75% in the fasted state when Angptl4 levels are naturally higher to inhibit LPL-mediated FA uptake, and by 80 and 81% in the refed state, respectively (*Figure 3B*). With this lower dosage, anti-Angptl4 and combined ASO treatment, but not anti-*Angptl3* ASO treatment, still increased liver weight (*Figure 3C*), explained by increased TG (see Supplementary material online, *Figure S4A*), and again mildly elevated plasma ALT activity (see Supplementary material online, *Figure S4B*). In this cohort, circulating levels of IFN-γ and TNF-α were elevated along with the gene expression of several pro-inflammatory markers in the liver following fasting but not refeeding with *Angptl4* and *Angptl3/4* silencing (see Supplementary material online, *Figure S4C*–*G*). In line with the short-term study, *Angptl3* or *Angptl4* silencing did not alter body weight (*Figure 3D*) or lean mass (*Figure 3E*), while *Angptl4* and combined *Angptl3/4* silencing decreased fat mass after 8 weeks (*Figure 3F*), and lowered the weights of gWAT and sWAT depots isolated after 12 weeks (*Figure 3G*) compared with negative ASO-treated mice. These effects were independent of total food intake, since *Angptl4* silencing tended to increase food intake from week 10 onwards and combined *Angptl3/4* silencing significantly increased food intake from week 4 onwards (*Figure 3H*, Supplementary material online, *Figure S4H*).

**Figure 4:**
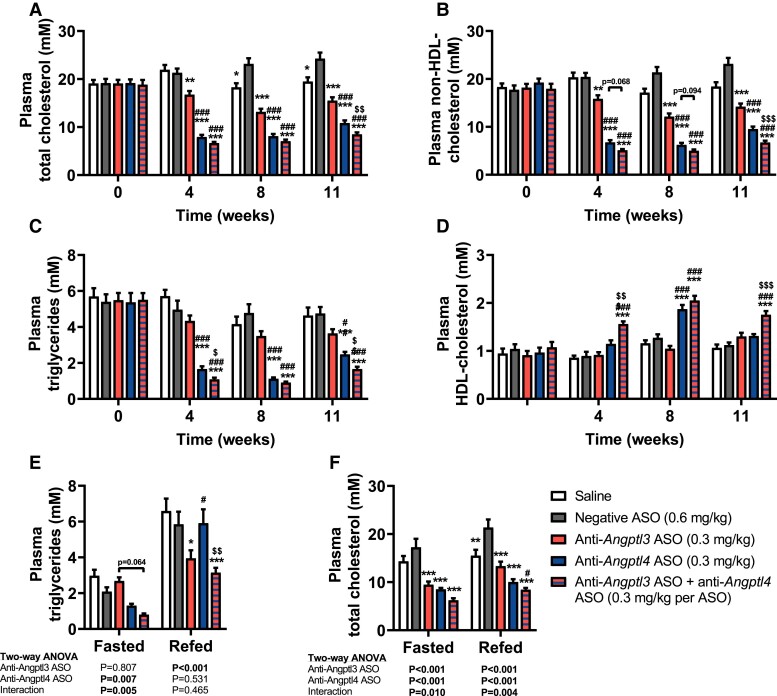
Long-term liver-targeted *Angptl3* and *Angptl4* silencing lowers plasma TG in refed and fasted state, respectively, in APOE*3-Leiden.CETP mice. Over a period of 12 weeks, female APOE*3-Leiden.CETP mice (8–14 weeks of age at the start of the study) were injected with saline (white) or the following hepatocyte-targeted antisense oligonucleotides (ASO): negative ASO (scrambled; 1.25 mg/kg, grey), anti-*Angptl3* ASO (0.3 mg/kg, red), anti-*Angptl4* ASO (0.3 mg/kg, blue), or anti-*Angptl3* ASO + anti-*Angptl4* ASO (0.3 mg/kg per ASO, red–blue striped). Levels of plasma (*A*) total cholesterol, (*B*) non-HDL-C, (*C*) triglycerides, and (*D*) HDL-C were measured at weeks 0, 4, 8, and 11, after 4 h of fasting (*n* = 12–16 per group). At week 12, levels of plasma (*E*) triglycerides and (*F*) total cholesterol were measured after 16 h of fasting (Fasted) or after 12 h of fasting and 4 h of refeeding (Refed) (*n* = 7–8 per group). The values are presented as means ± SEM. All groups were compared with each other, but the comparison with the saline group is not shown. ^*^any ASO group vs. negative ASO; ^#^any ASO group vs. anti-*Angptl3* ASO; ^$^anti-*Angptl4* ASO vs. anti-*Angptl3* ASO + anti-*Angptl4* ASO ^*,#,$^*P* < 0.05; ^**,##^*P* < 0.01; ^***,###,$$$^*P* < 0.001, according to two-way ANOVA on ranked values (*A, B, C*), two-way ANOVA (*D, E, F*), and following Tukey’s multiple comparison test. To discern anti-*Angptl3* ASO and anti-*Angptl4* ASO effects, extra two-way ANOVAs were performed per nutritional state for (*E, F*), in which saline was excluded; values are shown below the graphs.

We confirmed that *Angptl3* and *Angptl4* silencing lowered plasma TC and non-HDL-C levels in 4 h fasted mice (*Figure 4A* and *B*). In addition, 4 h fasted plasma TG levels were significantly lowered with *Angptl4* and combined *Angptl3/4* silencing (*Figure 4C*). Plasma HDL-C levels were elevated by combined *Angptl3/4* silencing throughout the study and by *Angptl4* silencing in week 8 when compared with negative ASO treatment (*Figure 4D*). Since endogenous ANGPTL3 and ANGPTL4 activity is highly dependent on nutritional status, plasma lipid levels were determined after 16 h fasting or after 12 h fasting followed by 4 h refeeding, at week 12. Accordingly, *Angptl3* silencing lowered plasma TG levels specifically after refeeding (*Figure 4E*; anti-*Angptl3* ASO effect by two-way ANOVA: *P* < 0.001), while *Angptl4* silencing lowered TG levels in the fasted state only (*Figure 4E*; anti-*Angptl4* ASO effect by two-way ANOVA: *P* = 0.007). Combined *Angptl3/4* silencing reduced plasma TG levels in both nutritional states, albeit non-significantly in the *post hoc* analysis (*Figure 4E*). In contrast to plasma TG levels, TC levels were consistently reduced by all ASO treatments in the fasted as well as the refed state (*Figure 4F*; anti-*Angptl3* ASO and anti-*Angptl4* ASO effects by two-way ANOVA: *P* < 0.001). These results suggest that the TG-lowering effects of liver-targeting *Angptl3* and *Angptl4* silencing are dependent on the nutritional state, while combined treatment consistently reduces plasma TG levels along with TC levels in various physiological settings.

### 3.5 Liver-targeted *Angptl3* and *Angptl4* silencing attenuates atherosclerosis development

**Figure 5:**
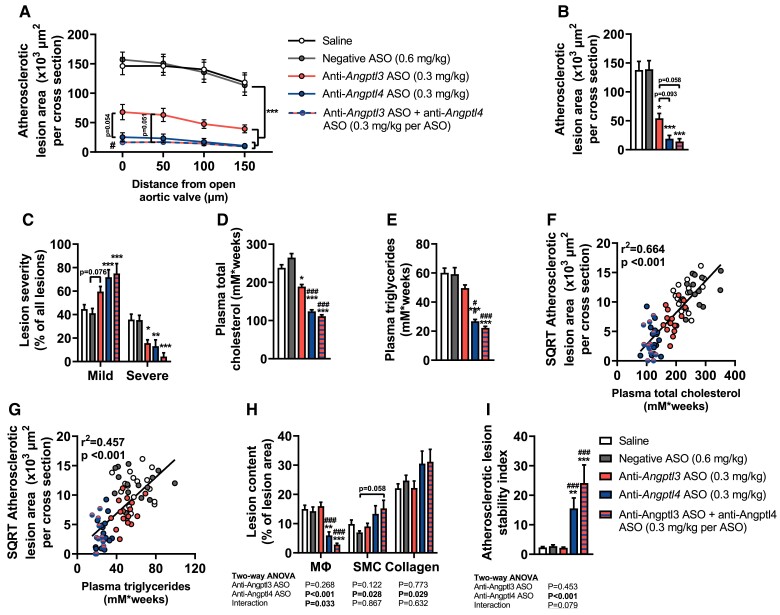
Long-term liver-targeted *Angptl3* and *Angptl4* silencing attenuates atherosclerosis development in APOE*3-Leiden.CETP mice. Over a period of 12 weeks, female APOE*3-Leiden.CETP mice (8–14 weeks of age at the start of the study) were injected with saline (white) or the following hepatocyte-targeted antisense oligonucleotides (ASO): negative ASO (scrambled; 1.25 mg/kg, grey), anti-*Angptl3* ASO (0.3 mg/kg, red), anti-*Angptl4* ASO (0.3 mg/kg, blue), or anti-*Angptl3* ASO + anti-*Angptl4* ASO (0.3 mg/kg per ASO, red–blue striped), after which mice were killed and their hearts were collected. Cross-sections of the aortic root area were stained with haematoxylin–phloxine–saffron, and (*A*) the atherosclerotic lesion area was determined and expressed as a function of distance from the appearance of open aortic valves and (*B*) the mean atherosclerotic lesion area was calculated (*n* = 15–16 per group). (*C*) Lesions were categorized according to lesion severity and expressed as a percentage of total lesions (*n* = 12–16 per group). (*D*) Total cholesterol and (*E*) triglyceride exposure during the study were determined by calculating the area under the curve and (*F, G*) plotted against the square root (SQRT) of the mean atherosclerotic lesion area from which B and Pearson correlation coefficients were determined (*n* = 13–16 per group). Cross-sections of the aortic root area were stained with anti-MAC3 antibody, anti-α-actin antibody, and Sirius Red to (*H*) quantify the content of macrophages (Mϕ), smooth muscle cells (SMC), and collagen, respectively, within the lesions (*n* = 15–16 per group). (*I*) Lesion stability index was calculated by dividing the sum of collagen and smooth muscle cell content by the macrophage content (*n* = 15–16 per group). The values are presented as means ± SEM (*n* = 12–16 per group). All groups were compared with each other, but the comparison with the saline group is not shown. ^*^Any ASO group vs. negative ASO; ^#^any ASO group vs. anti-*Angptl3* ASO; ^*,#^*P* < 0.05; ^**^P < 0.01; ^***,###^*P* < 0.001 according to one-way ANOVA (*A, D*) or (non-parametric) Kruskal–Wallis test (*B, C, E, H, I*), and following Šídák’s (*A, D*) or (non-parametric) Dunn’s (*B, C, E, H, I*) multiple comparison test, respectively. To discern anti-*Angptl3* ASO and anti-*Angptl4* ASO effects, extra two-way ANOVAs were performed for (*H, I*), in which saline was exclude; values are shown below the graphs.

In line with reduced plasma non-HDL-C levels, all treatments considerably reduced atherosclerotic lesion area throughout the aortic root (*Figure 5A* and *B*). In addition, all treatments decreased lesion severity, evident from a decline in the relative amount of severe lesions and an increase in the relative amount of mild lesions compared with the negative ASO group (*Figure 5C*). In this study, where the degree of *Angptl4* silencing was larger than that of *Angptl3*, *Angptl4* silencing consequently induced a greater attenuation of atherosclerosis development than *Angptl3* silencing, while the effects of *Angptl4* and combined *Angptl3/4* silencing were comparable. These effects related to cumulative TC and TG exposure throughout the study (*Figure 5D* and *E*), which correlated strongly with the average lesion area (*Figure 5F* and *G*), suggesting that the alleviation of atherosclerosis development by *Angptl3* and *Angptl4* silencing was driven by the reduction in hypocholesteraemia as well as hypertriglyceridaemia. Both *Angptl4* and combined *Angptl3/4* silencing, but not *Angptl3* silencing, strongly lowered the relative macrophage content of the lesions (*Figure 5H*; anti-*Angptl4* ASO effect by two-way ANOVA: *P* < 0.001) and tended to increase smooth muscle cell and collagen content (*Figure 5H*; anti-*Angptl4* ASO effect by two-way ANOVA: *P* = 0.028 and *P* = 0.029 for smooth muscle cell and collagen content, respectively). Together, these characteristics contributed to an increased lesion stability index after *Angptl4* and combined *Angptl3/4* silencing (*Figure 5I*), which tended to be even further elevated after combined *Angptl3/4* silencing compared with *Angptl4* silencing (*Figure 5I*; interaction effect by two-way ANOVA: *P* = 0.079). The representative pictures of all histological stainings of the atherosclerotic lesions are shown (see Supplementary material online, *Figure S5*).

## 4. Discussion

In contrast to ANGPTL3 inhibition, systemic inhibition of ANGPTL4 is not deemed a viable lipid-lowering pharmacological strategy due to the occurrence of adverse gastrointestinal effects.^18,20,23^ Selectively inhibiting hepatic *Angptl4* may be a strategy to reduce hyperlipidaemia and atherosclerosis without inducing these side effects.^28,29^ Here, we compared the therapeutic potential of a GalNAc-conjugated ASO that inhibits hepatic *Angptl4* with that of a liver-targeted anti-*Angptl3* ASO in APOE*3-Leiden.CETP mice, a hyperlipidemic and atherosclerosis-prone model for human-like lipoprotein metabolism. We demonstrate in this translational model that *Angptl4* silencing potently attenuated hyperlipidemia and atherosclerosis development.

Both *Angptl3* and *Angptl4* silencing, but not negative ASO treatment, caused enlargement of the liver as explained by increased hepatic TG. This is in line with a modest liver enlargement recently observed using the same anti-*Angptl4* ASO in C57BL/6J mice.^29^ Although liver lipids as well as inflammatory markers should be closely monitored in future clinical trials, importantly, we did not observe any hallmarks of ASO toxicity,^41,42^ and demonstrated that the administration of a GalNAc ASO targeting human *ANGPTL4* was well tolerated and induced no adverse effects in cynomolgus monkeys. Body weight was not changed by the ASO treatments as a non-significant reduction in fat mass compensated for the increased liver weight.

Our data point towards several potential mechanisms through which hepatic *Angptl4* silencing may attenuate hyperlipidaemia, and accordingly atherosclerosis development, which only partly overlap with those of *Angptl3* silencing. First, only *Angptl4* silencing elevated lipid uptake by BAT, and we previously demonstrated that activation of BAT attenuates hyperlipidaemia and atherosclerosis development by delipidating TRLs to subsequently accelerate hepatic TRL-remnant clearance.^35^ The elevated lipid uptake upon hepatic *Angptl4* silencing in our study also suggests that liver-derived ANGPTL4 acts systemically on BAT in addition to the autocrine and paracrine effects of ANGTPL4 in BAT.^13,14,26,43^ This effect on lipid uptake by BAT likely extends to other adipose tissues, as hepatic *Angptl4* silencing also enhanced LPL activity in WAT, which we were unfortunately unable to demonstrate here due to technical limitations.^29^ This is in line with genetic mimicry analysis in humans, suggesting that ANGPTL4 exerts its function through LPL.^44^ Second, only *Angptl4* silencing increased the ratio of hepatic uptake of TRL-derived oleate over TRL-derived cholesteryl oleate. In line with this finding, a previous study showed that genetic deletion of *Angptl4* in the liver increased hepatic lipase-dependent FA uptake by the liver in mice,^28^ but it remains unclear to what extent this contributes to lipid-lowering effects in humans and in this study. Third, hepatic *Angptl4* as well as *Angptl3* silencing largely reduced hepatic VLDL secretion, as explained by reduced ApoB production likely following transcriptional suppression. This is in line with global *Angptl4* deletion or ANGPTL4 inhibition by mAbs in a previous mouse study, albeit by an unknown mechanism.^23^ In contrast, hepatocyte-specific genetic deletion of *Angptl4* did not affect VLDL production in chow-fed C57BL/6J mice,^28,45^ suggesting that *Angptl4* silencing affects hyperlipidaemic and non-hyperlipidaemic models differently. The TG-lowering effects of *Angptl3* and *Angptl4* silencing only occurred during the refed and fasted state, respectively, indicating dependency on the nutritional state. The effects of *Angptl4* silencing on lipid uptake by BAT and VLDL secretion may therefore also be dependent on the nutritional state. Interestingly, combined *Angptl3* and *Angptl4* silencing reduced plasma TG levels independent of the nutritional state, opposed to the single ASO treatment, and may thereby provide added clinical benefits. In this mouse model of lipid-driven atherosclerosis development, the reduced atherosclerotic lesion formation was primarily a function of the reduced plasma lipid levels following *Angptl3* and *Angptl4* silencing. Furthermore, combined *Angptl3* and *Angptl4* silencing tended to further elevate the atherosclerotic lesion stability index, which is associated with lowered risk for plaque rupture and cardiovascular events in humans.^2,46^

Our findings in mice with a humanized lipoprotein profile suggest that liver-specific targeting of *ANGPTL4* holds therapeutic promise for the treatment of hyperlipidaemia and asCVD. Inhibition of hepatic *ANGPTL4* may therefore be a promising strategy to reduce plasma TG levels in addition to classical LDL-C-lowering therapies, a statement that finds support in comparative human genetic studies that demonstrate additional benefits of TG-lowering *LPL* variants in addition to LDL-C-lowering alleles on reduced asCVD risk.^47–49^ While *Angptl4* is expressed in a variety of tissues in mice, in humans, *ANGPTL4* is predominantly expressed in the liver,^15,50^ suggesting that lipid-lowering effects of hepatic *ANGPTL4* silencing may be more pronounced in humans than hepatic *Angptl4* silencing in mice. Our findings suggest that combining hepatic *ANGPTL3* and *ANGPTL4* silencing provides additive benefit by lowering plasma TG levels independent of the nutritional state. The human *ANGPTL4*-targeting GalNAc ASO Lipisense is currently advancing into Phase-II clinical testing, and the initial results of the Phase-I trial do not report any adverse effects.^51^

**Translational perspective**

Hyperlipidaemia, characterized by elevated plasma cholesterol and triglyceride levels, is the main driver of atherosclerotic cardiovascular disease. This study demonstrates a strong lipid lowering and anti-atherosclerotic effect of the liver-specific silencing of angiopoietin-like 4 (ANGPTL4), a modulator of lipoprotein metabolism, using antisense oligonucleotides in humanized mice. While pharmacological systemic inhibition of ANGPTL4 was previously observed to cause adverse effects, this novel liver-targeted treatment approach is well tolerated in non-human primates, and a liver-targeted antisense oligonucleotide against human ANGPTL4 is currently in clinical development.

## Supplementary Material

cvae195_Supplementary_Data

## Data Availability

The data supporting the findings of this study are available from the corresponding author upon reasonable request.
