# Supplementary material for: Liver-targeted Angptl4 silencing by antisense oligonucleotide treatment attenuates hyperlipidaemia and atherosclerosis development in APOE*3-Leiden.CETP mice
Source: Cardiovasc Res. 2024 Sep 11;120(17):2179–90. doi: 10.1093/cvr/cvae195 (PMC11687395; doi:10.1093/cvr/cvae195)
Supplement: cvae195_Supplementary_Data [file cvae195_supplementary_data.zip › In het Panhuis & Modder - Angptl4 ASO_supp_revised_clean.docx]

**Supplementary Files**

**Liver-targeted *Angptl4* silencing by antisense oligonucleotide treatment attenuates hyperlipidemia and atherosclerosis development in APOE*3-Leiden.CETP mice**

Modder & In het Panhuis et al.

**Diet composition**

Purified Diet T with 40.5% sucrose, 20% casein, 15% cacao butter, 1% corn oil and 0.15% cholesterol.

| **Crude nutrients** |  |  | **Carbohydrates** |  |  | **Trace elements** |  |
| --- | --- | --- | --- | --- | --- | --- | --- |
| Crude protein | 176.16 g/kg |  | Inositol | 499.98 mg |  | Iron | 129.98 mg |
| Crude fat | 160.45 g/kg |  | Starch | 78.50 g/kg |  | Manganese | 63.50 mg |
| Crude fiber | 62.69 g/kg |  | Sugars | 405.00 g/kg |  | Zinc | 52.40 mg |
| Sugar and starch | 495.16 g/kg |  | Lactose | 0.40 g/kg |  | Copper | 17.97 mg |
| N free extracts | 508.47 g/kg |  | Cellulose | 55.80 g/kg |  | Cobalt | 0.14 mg |
|  |  |  | Glucose | 3.70 g/kg |  | Iodine | 0.47 mg |
| **Fatty acids** |  |  |  |  |  | Selenium | 0.19 mg |
| C16:0 | 40.10 g/kg |  | **Minerals** |  |  | Chromium | 0.49 mg |
| C18:0 | 52.70 g/kg |  | Calcium | 7.12 g/kg |  | Nickel | 0.07 mg |
| C18:1 | 55.05 g/kg |  | Phosphorus | 4.17 g/kg |  | Fluorine | 2.12 mg |
| C18:2 | 10.50 g/kg |  | Potassium | 5.68 g/kg |  | Arsenic | 0.07 mg |
| C18:3 | 0.10 g/kg |  | Magnesium | 1.57 g/kg |  | Lead | 0.80 mg |
| C20-C22 | 1.51 g/kg |  | Sodium | 1.15 g/kg |  | Aluminium | 3.31 mg |
|  |  |  | Chlorine | 7.59 g/kg |  |  |  |
| **Amino acids** |  |  | Sulfur | 0.54 g/kg |  |  |  |
| Lysine | 12.42 g/kg |  |  |  |  |  |  |
| Methionine | 6.78 g/kg |  | **Vitamins** |  |  |  |  |
| Cystine | 0.52 g/kg |  | Vitamin A | 18.00 IU/g |  |  |  |
| Threonine | 7.12 g/kg |  | Vitamin D3 | 2.00 IU/g |  |  |  |
| Tryptophan | 2.32 g/kg |  | Vitamin E | 62.67 mg |  |  |  |
| Isoleucine | 10.08 g/kg |  | Vitamin K | 10.00 mg |  |  |  |
| Arginine | 6.12 g/kg |  | Thiamine (B1) | 20.00 mg |  |  |  |
| Phenylalanine | 7.28 g/kg |  | Riboflavin (B2) | 11.56 mg |  |  |  |
| Histidine | 4.64 g/kg |  | Pyridoxine (B6) | 15.33 mg |  |  |  |
| Leucine | 19.04 g/kg |  | Niacin | 39.20 mg |  |  |  |
| Tyrosine | 9.44 g/kg |  | Pantothenic acid | 15.90 mg |  |  |  |
| Valine | 11.76 g/kg |  | Cobalamin (B12) | 50.00 µg |  |  |  |
| Alanine | 3.96 g/kg |  | Folic acid | 7.84 mg |  |  |  |
| Aspartic acid | 9.26 g/kg |  | Choline | 7457.8 mg |  |  |  |
| Glutamic acid | 35.10 g/kg |  | Biotin | 306.65 µg |  |  |  |
| Glycine | 5.12 g/kg |  |  |  |  |  |  |
| Proline | 16.06 g/kg |  |  |  |  |  |  |

**Table S1. Primer sequences**

| Gene | Forward primer (5’-3’) | Reverse primer (5’-3’) |
| --- | --- | --- |
| *Adgre1* | CTTTGGCTATGGGCTTCCAGTC | GCAAGGAGGACAGAGTTTATCGTG |
| *Angptl3* | ACATGTGGCTGAGATTGCTGG | CCTTTGCTCTGTGATTCCATGTAG |
| *Angptl4* | GGAAAGAGGCTTCCCAAGAT | TCCCAGGACTGGTTGAAGTC |
| *Apob* | GCCCATTGTGGACAAGTTGATC | CCAGGACTTGGAGGTCTTGGA |
| *β2-microglobulin* | TGACCGGCTTGTATGCTATC | CAGTGTGAGCCAGGATATAG |
| *Cd68* | ATCCCCACCTGTCTCTCTCA | TTGCATTTCCACAGCAGAAG |
| *Cyp3a11* | CTTTCCTTCACCCTGCATTCC | CTCATCCTGCAGTTTTTTCTGGAT |
| *Hnf4* | AGAAGATTGCCAACATCAC | GGTCATCCAGAAGGAGTT |
| *Icam-1* | TCCGCTGTGCTTTGAGAACT | TCCGGAAACGAATACACGGT |
| *Il1b* | GCAACTGTTCCTGAACTCAACT | ATCTTTTGGGGTCCGTCAACT |
| *Mcp1* | GCATCTGCCCTAAGGTCTTCA | TTCACTGTCACACTGGTCACTCCTA |
| *Mttp* | CTCTTGGCAGTGCTTTTTCTCT | GAGCTTGTATAGCCGCTCATT |
| *Nr2f2* | CCTCAAAGTGGGCATGAGAC | TGGGTAGGCTGGGTAGGAG |
| *Sort1* | CCAAATGGGGACCAAACAACA | TCTCCATAATTCCAGGGCACC |
| *Tnfa* | AGCCCACGTCGTAGCAAACCAC | TCGGGGCAGCCTTGTCCCTT |
| *Vcam-1* | TGGAGGTCTACTCATTCCCTGA | GACAGGTCTCCCATGCACAA |
| *Xbp1* | CTGAGTCCGAATCAGGTGCAG | GTCCATGGGAAGATGTTCTGG |

**Table S2. Liver weight of cynomolgus monkeys**

|  | **Males** | | | | **Females** | | | |
| --- | --- | --- | --- | --- | --- | --- | --- | --- |
| **Group** | **1** | **2** | **3** | **4** | **1** | **2** | **3** | **4** |
| **Dose (mg/kg/dose)** | **0** | **3** | **10** | **30** | **0** | **3** | **10** | **30** |
| **N size** | **3** | **3** | **3** | **3** | **3** | **3** | **3** | **3** |
| Liver weight (including gallbladder) (g) | 68.8±6.7 | 61.5±1.1 | 67.2±5.6 | 80.4±4.5 | 66.0±13.2 | 59.2±3.8 | 56.8±9.6 | 55.1±5.3 |

**Table S3. Hepatic microscopic findings in cynomolgus monkeys**

|  | **Males** | | | | **Females** | | | |
| --- | --- | --- | --- | --- | --- | --- | --- | --- |
| **Group** | **1** | **2** | **3** | **4** | **1** | **2** | **3** | **4** |
| **Dose (mg/kg/dose)** | **0** | **3** | **10** | **30** | **0** | **3** | **10** | **30** |
| **No. animals examined** | **3** | **3** | **3** | **3** | **3** | **3** | **3** | **3** |
| **Livers (No. examined)** | (3) | (3) | (3) | (3) | (3) | (3) | (3) | (3) |
| Basophilic granules, hepatocellular (all of minimal severity) | 0 | 0 | 1 | 1 | 0 | 0 | 1 | 3 |
| Basophilic granules, Kupffer cell, (of minimal severity) | 0 | 0 | 0 | 0 | 0 | 0 | 0 | 1 |

**Table S4. Liver toxicity markers in cynomolgus monkeys.** AST, aspartate transaminase. ALT, alanine transaminase. *p<0.05, according to one-way ANOVA and following Dunn’s multiple-comparison test.

|  | **Males** | | | | **Females** | | | |
| --- | --- | --- | --- | --- | --- | --- | --- | --- |
| **Group** | **1** | **2** | **3** | **4** | **1** | **2** | **3** | **4** |
| **Dose (mg/kg/dose)** | **0** | **3** | **10** | **30** | **0** | **3** | **10** | **30** |
| **N size** | **5** | **3** | **3** | **5** | **5** | **3** | **3** | **5** |
| **Day -12** |  | | | | | | | |
| AST (U/L) | 40.0±5.8 | 64.0±24.3 | 44.3±10.1 | 37.4±6.5 | 36.4±6.9 | 36.0±6.0 | 55.7±11.0* | 48.6±7.4 |
| ALT (U/L) | 46.4±10.5 | 90.3±64.7 | 44.0±4.6 | 50.4±6.6 | 45.8±10.4 | 41.0±2.0 | 47.0±13.5 | 52.4±9.6 |
| **Day 25** |  | | | | | | | |
| AST (U/L) | 34.0±3.9 | 37.7±4.6 | 25.0±5.6 | 29.8±5.5 | 30.6±3.2 | 25.0±4.6 | 28.0±1.7 | 32.2±10.8 |
| ALT (U/L) | 42.6±11.1 | 56.0±18.7 | 39.0±3.6 | 65.4±20.4 | 42.4±10.0 | 44.0±10.0 | 40.7±5.5 | 52.0±16.9 |

**Figure S1. Liver-targeted *Angptl3* and *Angptl4* silencing modulates energy metabolism.** Over a period of two weeks, female APOE*3-Leiden.CETP mice were injected five times with saline (white) or the following hepatocyte-targeted antisense oligonucleotides (ASO): negative ASO (scrambled; 1.25 mg/kg, grey), anti-Angptl3 ASO (1.25 mg/kg, red), anti-Angptl4 ASO (1.25 mg/kg, blue), or anti-Angptl3 ASO + anti-Angptl4 ASO (1.25 mg/kg per ASO, red-blue striped). At endpoint, **(A)** expression of Angptl4 was measured in gastrocnemius (Gastr), interscapular brown adipose tissue (iBAT), and gonadal white adipose tissue (gWAT), (n=8 per group). During the last eight days, mice were single-housed in metabolic cages where **(B)** energy expenditure, **(C)** fat oxidation rate, and **(D)** carbohydrate oxidation rate were assessed via indirect calorimetry (n=6 per group). Values in **(A)** are presented as geometric means ± 95% confidence interval and values in **(B-D)** as means ± SEM. * any group vs. negative ASO; ^#^ any group vs. anti-Angptl3 ASO; ^#,$^ p<0.05 according to two-way ANOVA and following Tukey’s multiple comparisons tests.

**Figure S2. Effect of liver-targeted *Angptl3* and *Angptl4* silencing on adipose tissue LPL.** Over a period of two weeks, female APOE*3-Leiden.CETP mice were injected five times with saline or the following hepatocyte-targeted antisense oligonucleotides (ASO): negative ASO (scrambled; 1.25 mg/kg), anti-Angptl3 ASO (1.25 mg/kg), anti-Angptl4 ASO (1.25 mg/kg), or anti-Angptl3 ASO + anti-Angptl4 ASO (1.25 mg/kg per ASO). Representative Western blots of the quantification of LPL in gWAT are shown along with a Ponceau S staining of the membrane showing total protein loading that was used for the normalization of the LPL bands.

**Figure S3. Liver-targeted *Angptl3* and *Angptl4* silencing induces hypertrophy of hepatocytes without causing inflammation in APOE*3-Leiden.CETP mice.** Over a period of two weeks, female APOE*3-Leiden.CETP mice were injected five times with saline (white) or the following hepatocyte-targeted antisense oligonucleotides (ASO): negative ASO (scrambled; 1.25 mg/kg, grey), anti-Angptl3 ASO (1.25 mg/kg, red), anti-Angptl4 ASO (1.25 mg/kg, blue), or anti-Angptl3 ASO + anti-Angptl4 ASO (1.25 mg/kg per ASO, red-blue striped). At endpoint, **(A)** liver weight was determined (n=8 per group). Cross-sections of the liver were stained with hematoxylin & eosin (H&E), an antibody against macrophage-marker F4/80 and collagen-staining Sirius red. **(B)** F4/80 and **(C)** collagen content were quantified and **(D)** representative pictures are shown. **(E)** Hepatic gene expression was measured of macrophage markers [cluster of differentiation 68 (*Cd68*)*,* adhesion G Protein-Coupled Receptor E1 *(Adgre*)*,* monocyte chemoattractant protein-1 (*Mcp-1*)], inflammatory markers [tumor necrosis factor alpha (*Tnf-a*), interleukin-1b (*Il-1b*)], leucocyte recruitment markers [vascular cell adhesion protein 1 (*Vcam-1*) and intercellular Adhesion Molecule 1 (*Icam-1*)] (n=6-8 per group). In plasma, **(F)** alanine transaminase activity was determined (n=8 per group). At week 2, levels of plasma (G) interferon-γ (IFN-γ), **(H)** Il-1β, **(I)** TNFα. Values are presented as means ± SEM. * any group vs. negative ASO; ^#^ any group vs. anti-Angptl3 ASO; ^$^ anti-Angptl4 ASO vs. anti-Angptl3 ASO + anti-Angptl4 ASO ^#,$^ p<0.05; ^,##^ p<0.01; ***^,###^ p<0.001, according to one-way ANOVA **(A, D)**, Kruskal-Wallis test **(F)** and following Tukey’s **(A, D)** or Dunn’s **(F)** multiple-comparison test.

**Figure S4. Long-term liver-targeted *Angptl3* and *Angptl4* silencing alters hepatic lipid content in APOE*3-Leiden.CETP mice.** Over a period of 12 weeks, female APOE*3-Leiden.CETP mice were injected with saline (white) or the following hepatocyte-targeted antisense oligonucleotides (ASO): negative ASO (scrambled; 1.25 mg/kg, grey), anti-Angptl3 ASO (0.3 mg/kg, red), anti-Angptl4 ASO (0.3 mg/kg, blue), or anti-Angptl3 ASO + anti-Angptl4 ASO (0.3 mg/kg per ASO, red-blue striped). At endpoint, **(A)** the hepatic content of triglycerides was measured (n=7-8 per group), and **(B)** plasma alanine transaminase activity was determined (n=13-16 per group). Plasma levels of the cytokines IFN-γ **(C)**, IL-1β **(D)** and TNF-α were measured. Gene expression of macrophage markers, inflammatory markers and leukocyte recruitment markers was measured in the liver of 16 hour fasted **(F)** and **(G)** 12 hours fasted and 4 hours refed mice. **(H)** Food intake was monitored and expressed as cumulative food intake per mouse (n=4 cages of three to four mice per cage). Values are presented as means ± SEM. * any group vs. negative ASO; ^#^ any group vs. anti-Angptl3 ASO; ^$^ anti-Angptl4 ASO vs. anti-Angptl3 ASO + anti-Angptl4 ASO ***^,#,$^** p<0.05; ****^,##^** p<0.01; *****^,###^** p<0.001, according to Kruskal-Wallis test **(A)** or two-way ANOVA **(B, C)** and following Dunn’s **(A)** or Tukey’s **(C)** multiple comparisons tests.

**Figure S5. Long-term liver-targeted *Angptl3* and *Angptl4* silencing attenuates atherosclerosis development in APOE*3-Leiden.CETP mice.** Over a period of 12 weeks, female APOE*3-Leiden.CETP mice were injected with saline or the following hepatocyte-targeted antisense oligonucleotides (ASO): negative ASO (scrambled; 1.25 mg/kg), anti-Angptl3 ASO (0.3 mg/kg), anti-Angptl4 ASO (0.3 mg/kg), or anti-Angptl3 ASO + anti-Angptl4 ASO (0.3 mg/kg per ASO), after which mice were killed and hearts were collected. Cross-sections of the aortic root area were stained with hematoxylin-phloxine-saffron, antibodies against MAC3 and α-actin and collagen-staining Sirius red. **(A)** Representative pictures are shown (n=15-16 per group).
