# Supplementary material for: Liver-targeted Angptl4 silencing by antisense oligonucleotide treatment attenuates hyperlipidaemia and atherosclerosis development in APOE*3-Leiden.CETP mice
Source: Cardiovasc Res. 2024 Sep 11;120(17):2179–90. doi: 10.1093/cvr/cvae195 (PMC11687395; doi:10.1093/cvr/cvae195)
Supplement: cvae195_Supplementary_Data [file cvae195_supplementary_data.zip › MS_Figure S5.pdf]

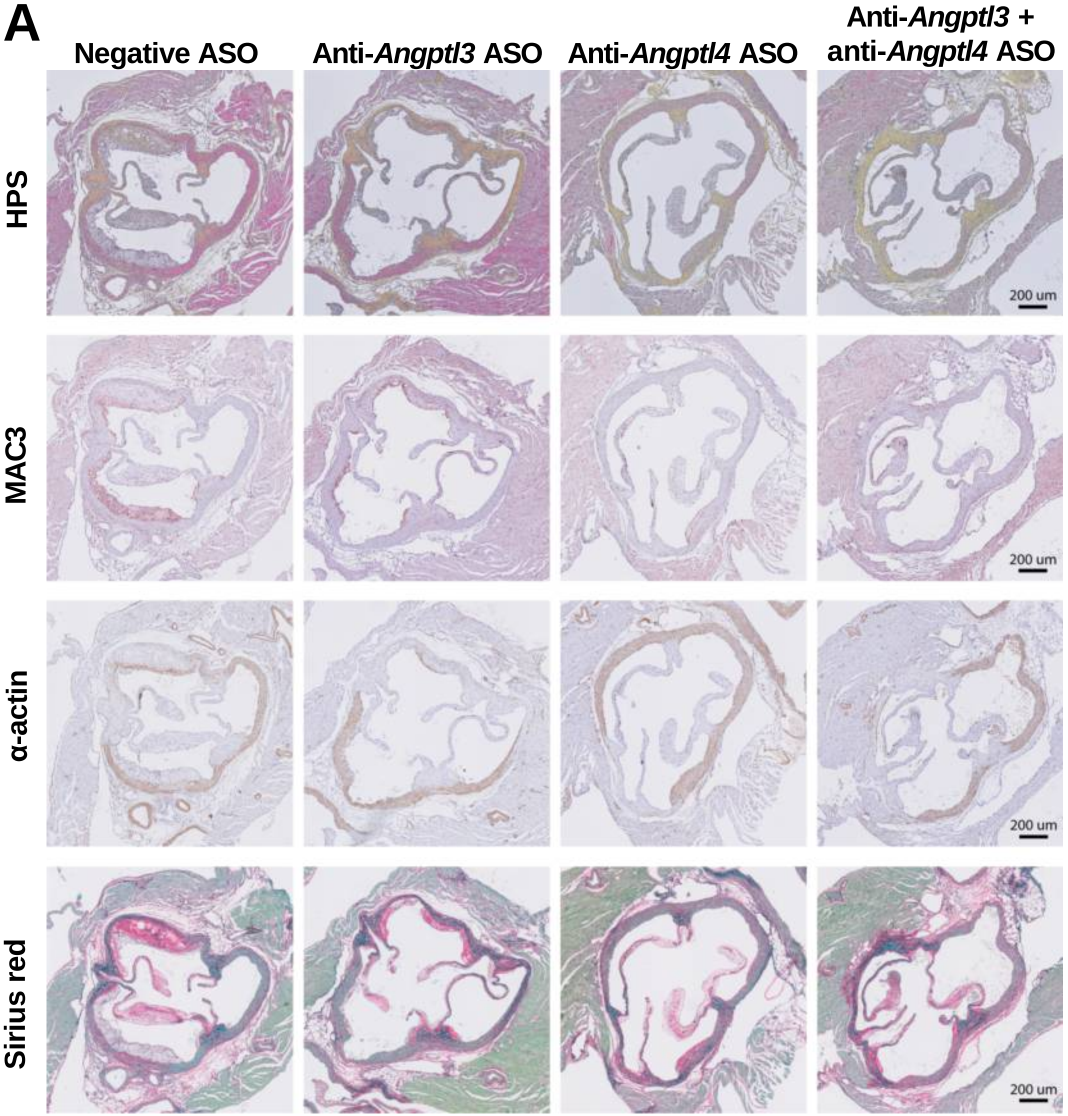
A
Negative ASO
Anti-Angptl3 ASO
Anti-Angptl4 ASO
Anti-Angptl3 + anti-Angptl4 ASO
HPS
MAC3
α-actin
Sirius red
200 um
200 um
200 um
200 um
