# Supplementary figures and images for: Liver-targeted Angptl4 silencing by antisense oligonucleotide treatment attenuates hyperlipidaemia and atherosclerosis development in APOE*3-Leiden.CETP mice
Source: Cardiovasc Res. 2024 Sep 11;120(17):2179–90. doi: 10.1093/cvr/cvae195 (PMC11687395; doi:10.1093/cvr/cvae195)

**A**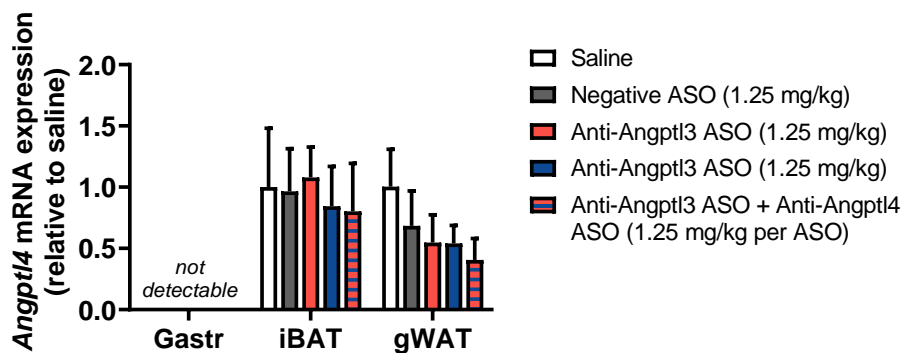**B**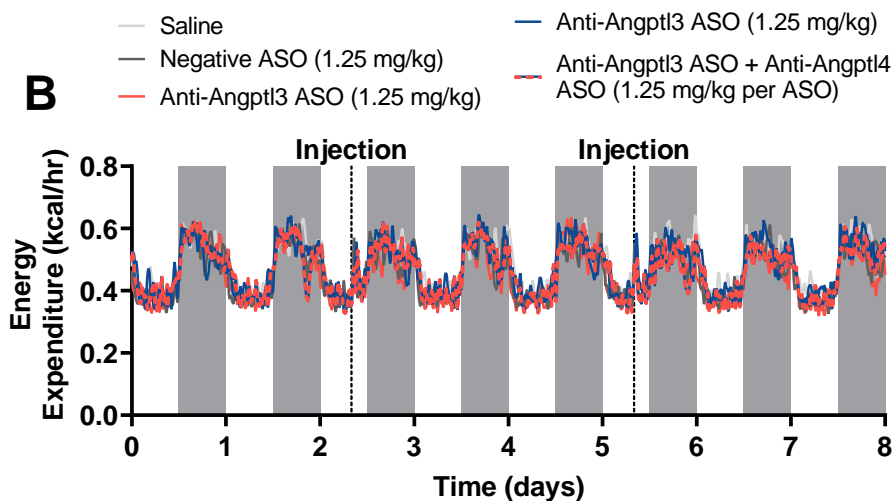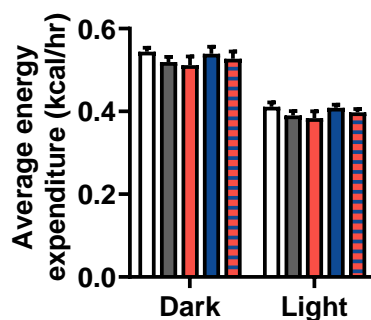**C**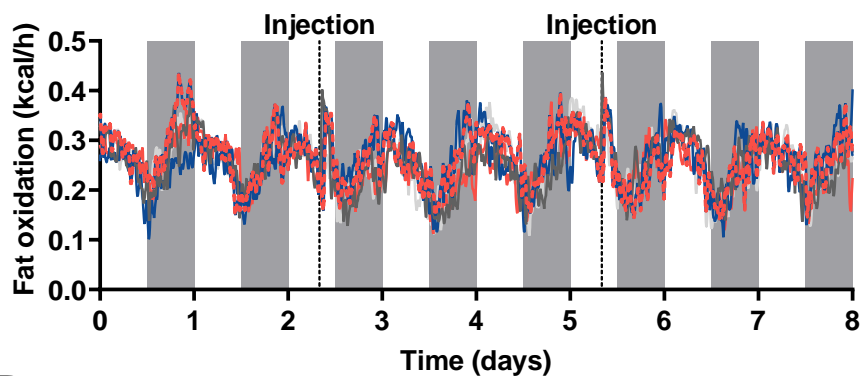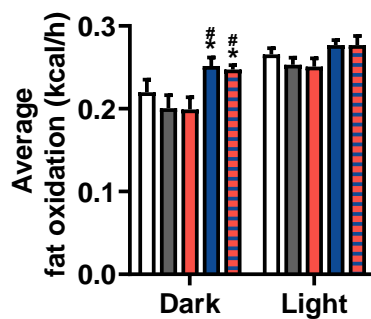**D**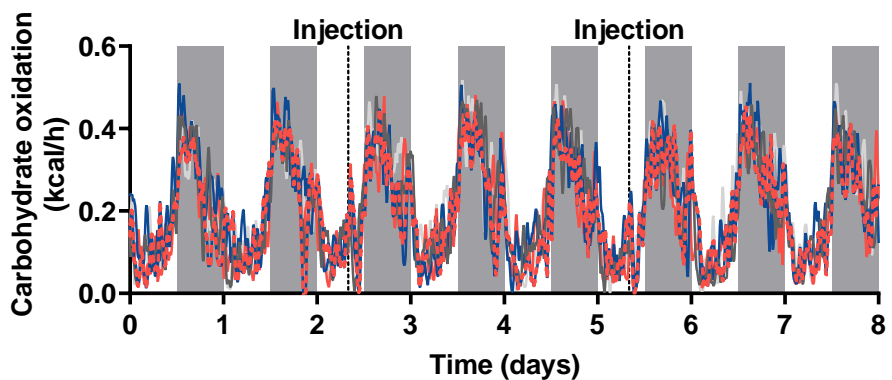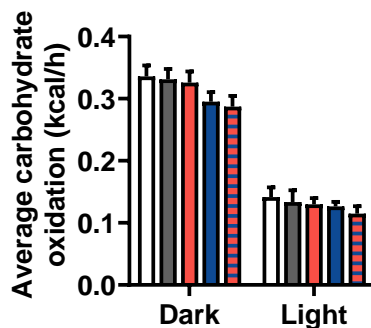

Supplement: cvae195_Supplementary_Data [file cvae195_supplementary_data.zip › MS_Figure S1.pdf]

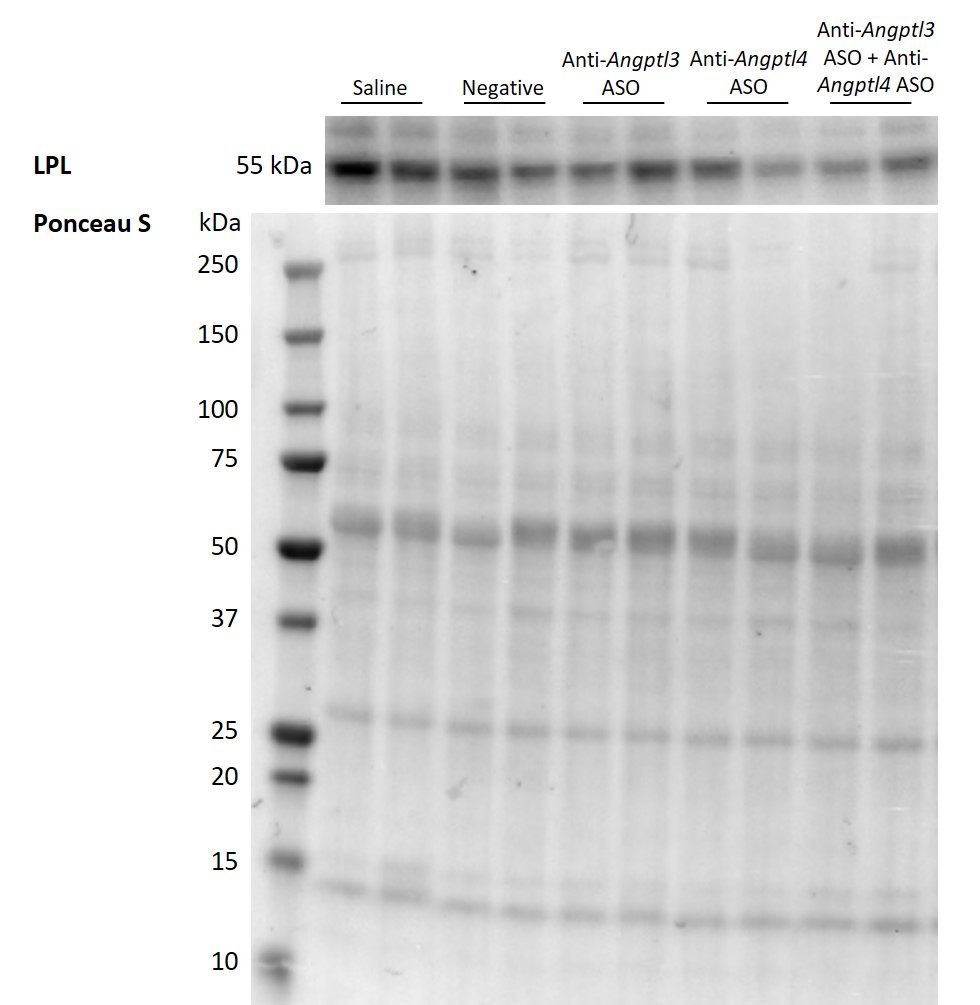

Supplement: cvae195_Supplementary_Data [file cvae195_supplementary_data.zip › MS_Figure S2.jpg]

**A**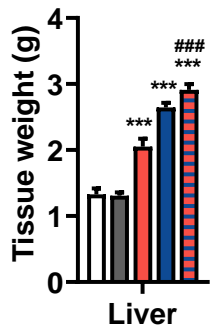**B**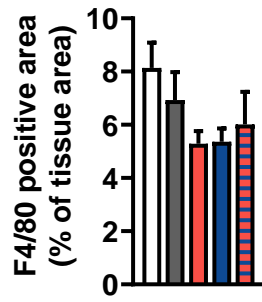**C**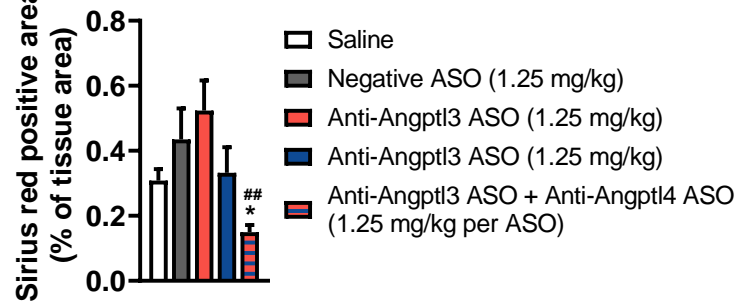**D**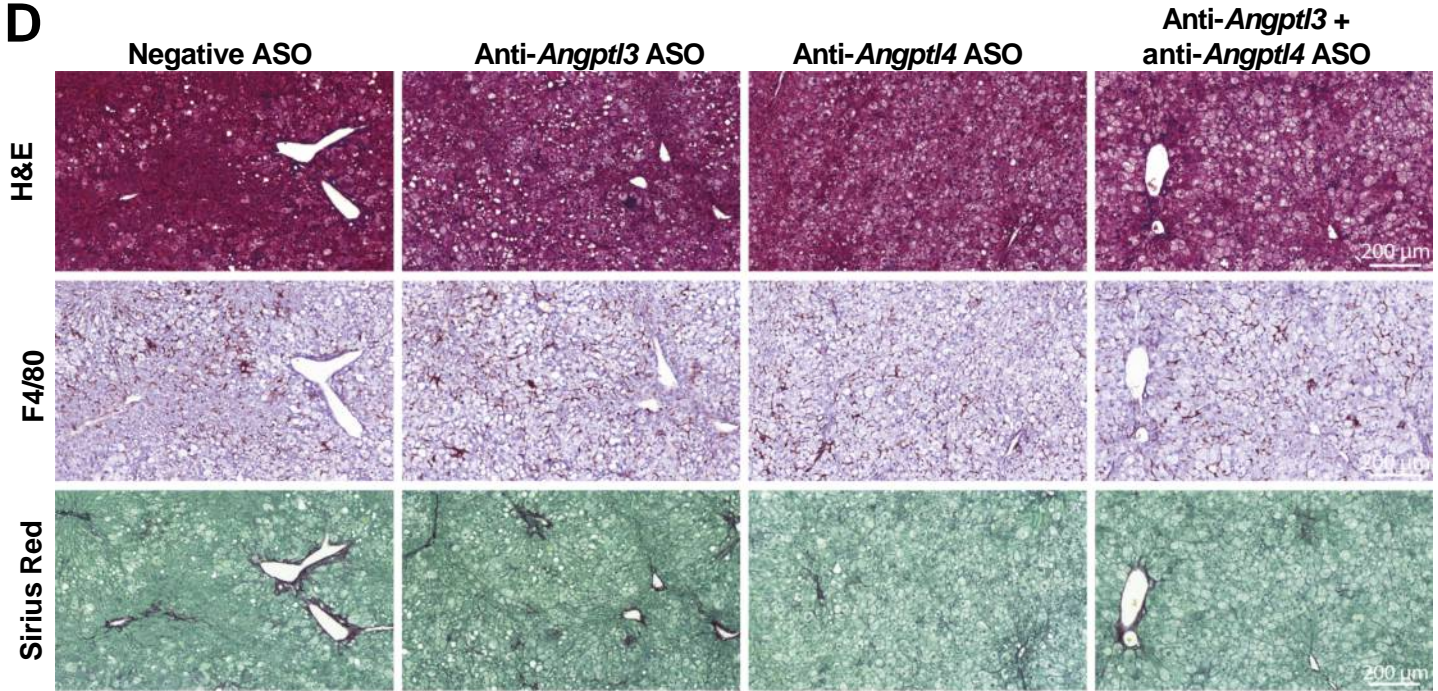**E**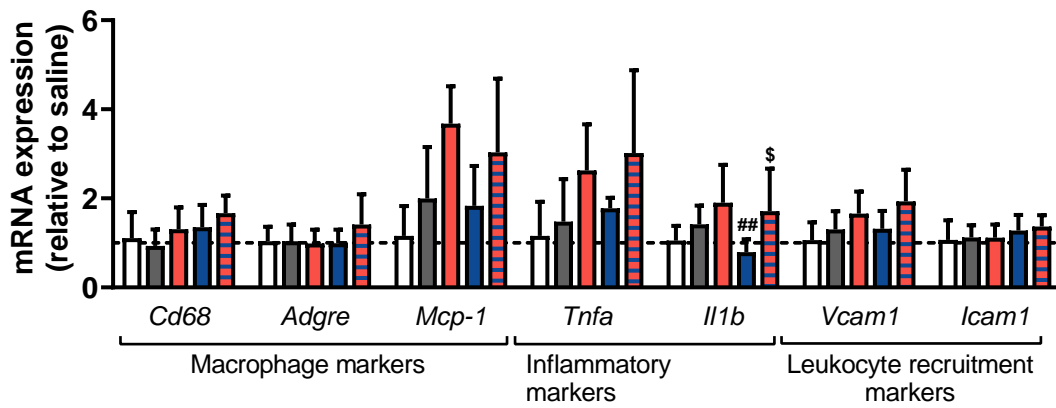**F**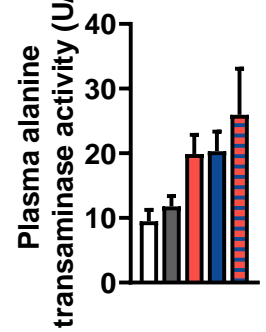**G**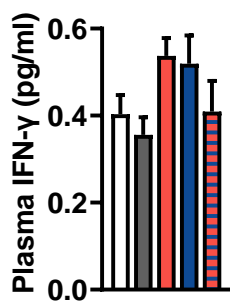**H**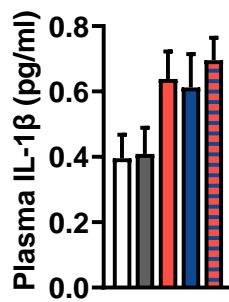**I**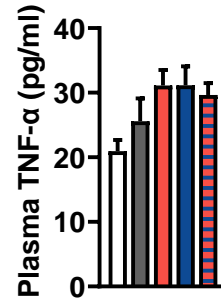

Supplement: cvae195_Supplementary_Data [file cvae195_supplementary_data.zip › MS_Figure S3.pdf]

**A**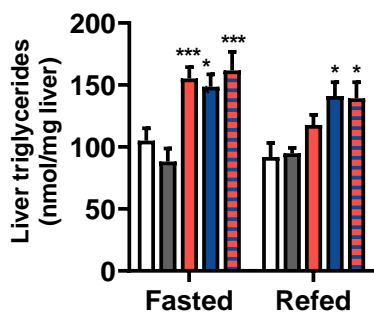**B**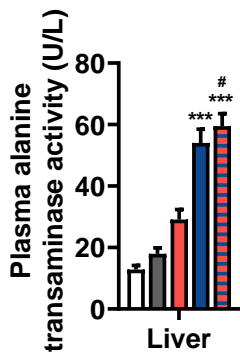**C**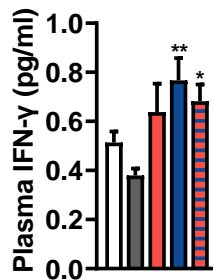**D**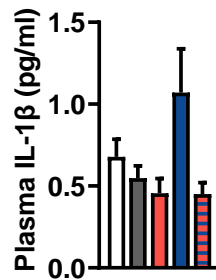**E**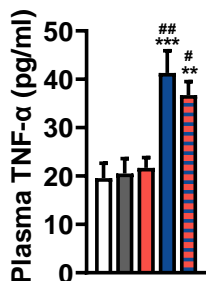**F**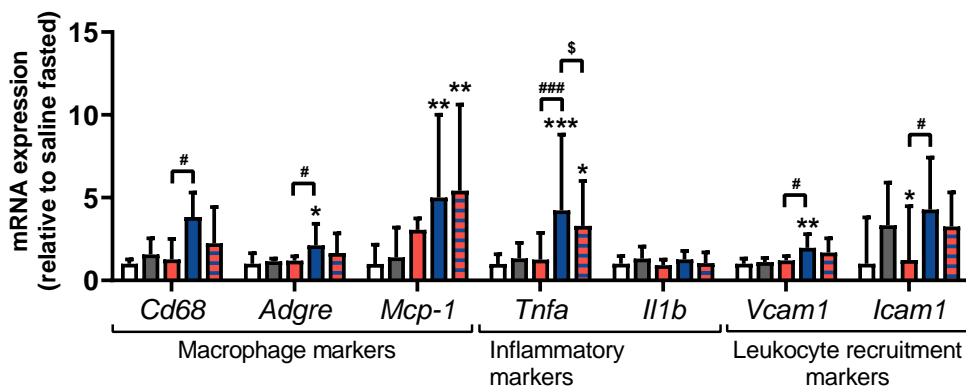**G**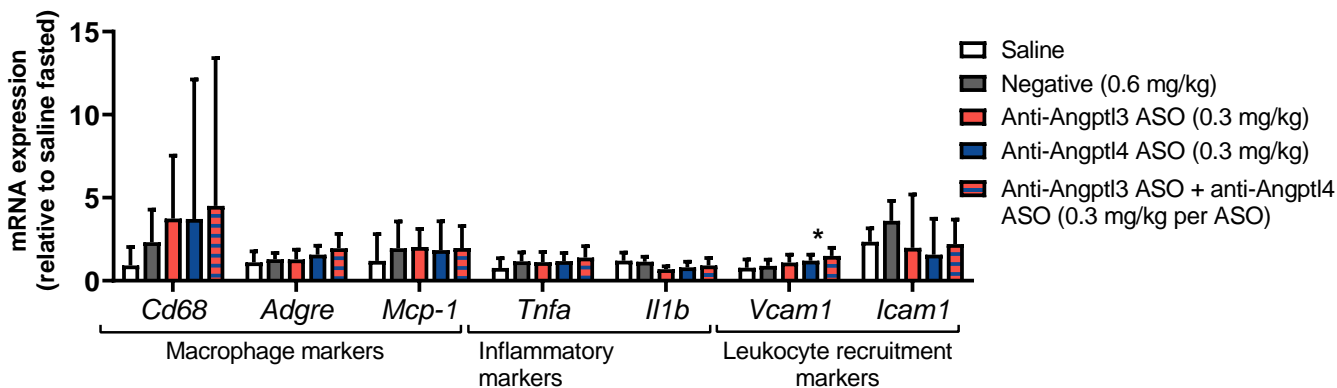**H**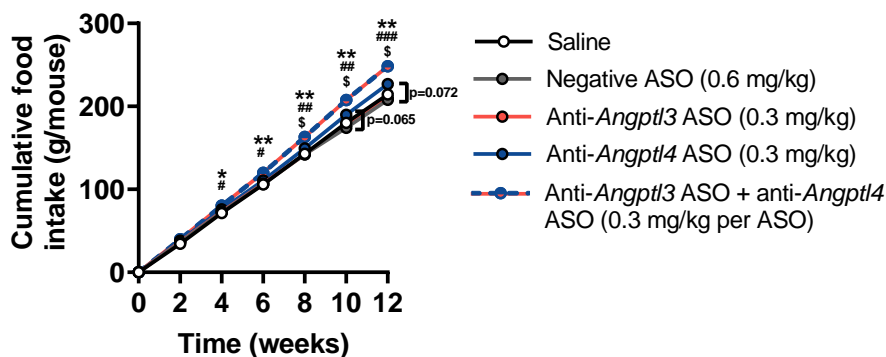

Supplement: cvae195_Supplementary_Data [file cvae195_supplementary_data.zip › MS_Figure S4.pdf]
